# Supplementary material for: Exploring Structure-Property Relationships in a Bio-Inspired Family of Bipodal and Electronically-Coupled Bistriphenylamine Dyes for Dye-Sensitized Solar Cell Applications
Source: Molecules. 2020 May 11;25(9):2260. doi: 10.3390/molecules25092260 (PMC7248778; doi:10.3390/molecules25092260)
Supplement: Supplementary file 1 [file molecules-25-02260-s001.pdf]

# Exploring structure-property relationships in a bio-inspired family of bipodal and electronically-coupled *bis*triphenylamine dyes for dye-sensitized solar cell applications

Tamara Al-Faouri, Francis L. Buguis, Saba Azizi-Soldouz, Olga V. Sarycheva, Burhan A. Hussein, Reeda Mahmood, Bryan D. Koivisto*[a]

**Table of Contents**

1. General Considerations ........ 1
2. Synthesis & Experimental ........ 2
3. Summary of Physicochemical Characterization ........ 18
4. UV-Vis and Fluorescence Spectroscopy ........ 19
   UV-Vis in DCM ........ 19
   Absorption and Fluorescence in DCM ........ 20
5. Cyclic Voltammetry ........ 23
6. IV Curves ........ 28
7. References ........ 30

# 1. General Considerations

All reagents were purchased from Aldrich except palladium complexes which were purchased from Pressure Chemical Co. (Pittsburg, PA). Purification by column chromatography was carried out using silica (Silicycle: ultrapure flash silica). Analytical thin-layer chromatography was performed on aluminum-backed sheets precoated with silica 60 F254 adsorbent (0.25 mm thick; Silicycle) and visualized under UV light. Routine $^{1}H$, $^{13}C\{^{1}H\}$, NMR spectra were recorded at 400 and 100 MHz, respectively, on a Bruker AV 400 instrument at ambient temperature. Chemical shifts (δ) are reported in parts per million (ppm) from down to up-field and referenced to a residual nondeuterated solvent ($CHCl_3$; 7.26 ppm for 1H & 77.1 ppm for 13C). *$^{13}C$ were not able to be recorded for the biscyanoacetic acid dyes because of a lack of solubility.* Standard abbreviations indicating multiplicity are used as follows: s = singlet; d = doublet; m = multiplet; br = broad. High resolution mass spectroscopy (HRMS) results were obtained from Queens University (Kingston, Canada) or Ulm University (Ulm, Germany – MALDI only). Electron impact (EI) mass spectrometry and Electrospray ionization (ESI) techniques were used for the ionization; time of flight (TOF) was used for analysis. All physicochemical measurements where performed in dichloromethane, unless otherwise noted. Cyclic voltammetry (CV) data was collected using a MetrOhm-Autolab Type II potentiostat/galvanostat. All CV were ran at 100 mV/s, in 0.1 M $NBu_4PF_6$, at a dye concentration of ~2 mM using a Pt working electrode and counter electrode, and a Ag wire pseudoreference, followed by referencing to an internal standard either OFc (octamethylferrocene: 225 mV vs NHE) or Fc (ferrocene: 700 mV vs NHE) depending on analyte wave overlap. UV-Vis absorption profiles were collected using an Agilent Cary 5000 UV-vis-NIR spectrophotometer. Fluorescence was measured with a Perkin Elmer LS-50B Luminescence Spectrometer. DFT calculations were performed using Gaussian16 Revision C.01. Naming of compounds as done using ChemDraw Professional v.16.

**Cell Fabrication.** Photoanodes were fabricated by screen-printing methods on fluorine-doped tin-oxide [FTO; Sigma Aldrich; TEC7 (7 Ω $cm^{-2}$)] using 2 layers of 18NR-T (20 nm particles, 12 μm thick), and 1 layer of WER4-O (100 nm particles, 6 μm thick) for a total thickness of 18 μm. The FTO glass was treated with $TiCl_4$(aq) (0.05 M) at 70 ℃ for 30 min and subsequently rinsed with $H_2O$ and EtOH. $TiO_2$ paste was applied to FTO glass and was air dried. Then it was heated in an oven at 125 ℃ for 6 min. Prior to coating with dye, $TiO_2$ substrates were treated with $TiCl_4$(aq) (0.05 M) at 70 ℃ for 30 min and subsequently rinsed with $H_2O$ and EtOH, then dried prior to heating. The electrodes were heated to 350 ℃ for 10 min, 450 ℃ for 15 min, and 500 ℃ for 15 min and left to cool to 80 ℃ prior to immersing into a DCM solution containing the dye (0.25 mM) for 16 h. The stained films were then rinsed with copious amounts of DCM and dried. The cells were fabricated using Pt-coated counter-electrode [FTO TEC-15 (15 Ω $cm^{-2}$)] and sealed with a 30 μm Surlyn (Dupont) gasket by resistive heating. The **Z1137** electrolyte used for this study was $I_3^-/I^-$ [1.0 M 1,3- dimethylimidazolium iodide (DMII), 60 mM $I_2$, 0.5 M *tert*-butylpyridine, 0.05 M NaI and 0.1 M GuNCS in acetonitrile. The electrolyte was introduced into the two-sandwiched electrodes via vacuum backfilling through a hole in the counter electrode. In the cases where the $I_3^-/I^-$ electrolyte was used, the hole was sealed with a 1x1 cm Surlyn sheet and a glass cover slip. The active area of the $TiO_2$ was 1 $cm^2$. Silver bus bars were added to all cells after sealing. Devices were tested with a mask of 0.25 $cm^2$ using a ScienceTech Xe Solar simulator with SciRunIV software connected to a Kiethley 2400 Source meter. Electrochemical Impedance Spectroscopy was performed using a MetrOhm-Autolab Type III potentiostat/galvanostat equipped with an FRA2 processor.

# 2. Synthesis & Experimental

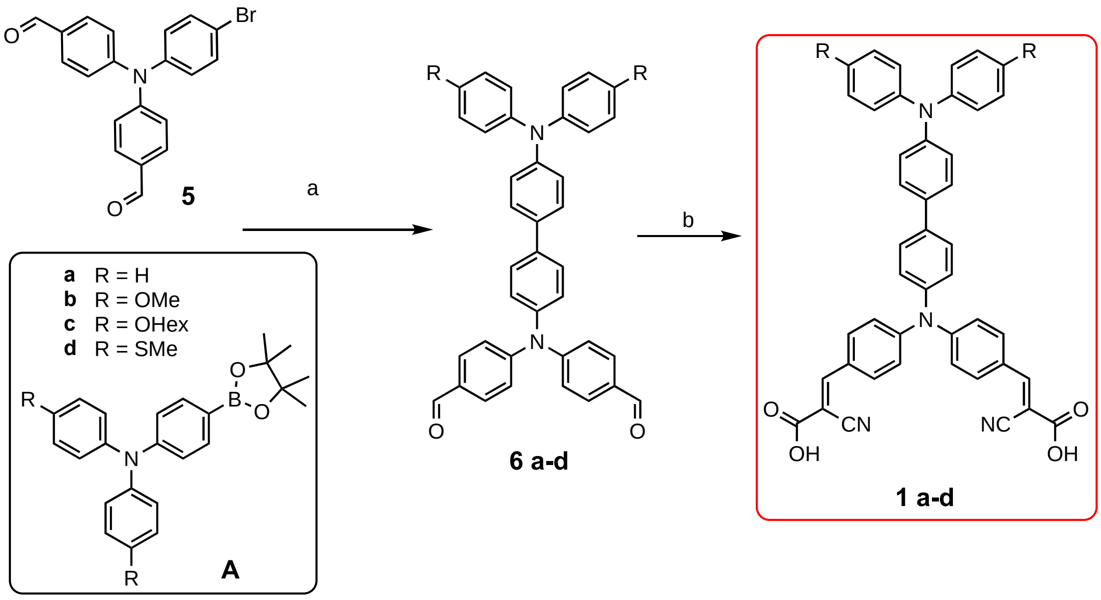


**Scheme S1.** Synthesis of aldehyde family **6 a-d** and dye family **1a-d**. Reaction conditions: a) **A** (1.1 eq.), $K_3PO_4$ (3.3 eq.), $Pd_2(dba)_3$ (2 or 4 mol %), [(t-Bu)$_3$PH]$BF_4$ (1 or 2 mol %), THF:$H_2O$ (9:1 v/v), reflux 16 h. b) cyanoacetic acid (6.0 eq.), piperidine (0.100 mL), $CHCl_3$:Hex (1:1 v/v), reflux 16 h.

Molecules **Aa,**[1] **Ab,**[2] **Ac,**[3] **Ad,**[4] **5,**[1] **6a,**[1] **6b,**[5] **1b**[5] have been previously reported

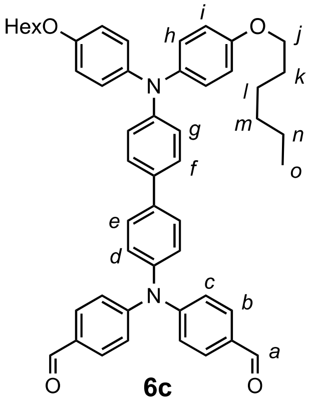


4,4'-((4'-(bis(4-(hexyloxy)phenyl)amino)-[1,1'-biphenyl]-4-yl)azanediyl)dibenzaldehyde (**6c**): In a 250 mL RBF with a magnetic stir bar, **5** (0.166 g, 0.44 mmol) and $K_3PO_4$ (0.336 g, 1.59 mmol) were dissolved in 20 mL of THF:$H_2O$ (9:1 v/v) and the mixture was sparged with $N_2$ for 30 minutes. The bisOHex-TPA-BPin (**A**c, 0.288 g, 0.480 mmol), $Pd_2(dba)_3$ catalyst (0.009 g, 0.009 mmol), and [(*t*-Bu)$_3$PH]$BF_4$ ancillary ligand (0.010 g, 0.035 mmol) were added and the mixture was heated to reflux under $N_2$ overnight (~ 12 h). The mixture was concentrated *in vacuo*. DCM (25 mL) was added and the solution was washed with water (3 x 50 mL). The organics were collected, washed with brine, dried over $MgSO_4$ and concentrated *in vacuo*. The residue was purified by silica gel column chromatography with Hex:EtOAc (3:1 v/v) as the eluent to afford **6c** (232 mg, 0.30 mmol, 68.2%). $^1$H NMR (400 MHz, $CDCl_3$): δ = 9.90 (H*a*, s, 2H), 7.79

(H*b*, d, $^3J_{HH}$ = 8 Hz, 4H), 7.55 (H*e*, d, $^3J_{HH}$ = 8 Hz, 2H), 7.40 (H*f*, d, $^3J_{HH}$ = 8 Hz, 2H), 7.23 (H*c*, d, $^3J_{HH}$ = 8 Hz, 4H), 7.18 (H*d*, d, $^3J_{HH}$ = 8 Hz, 2H), 7.08 (H*h*, d, $^3J_{HH}$ = 8 Hz, 4H), 6.98 (H*g*, d, $^3J_{HH}$ = 8 Hz, 2H), 6.84 (H*i*, d, $^3J_{HH}$ = 8 Hz, 4H), 3.94 (H*j*, t, $^3J_{HH}$ = 8 Hz, 4H), 1.78 (H*k*, dt, 4H), 1.46 (H*l*, dt, 4H), 1.35 (H*m*,H*n*, m, 8H), 0.92 (H*o*, t, 6H). $^{13}C\{^1H\}$ NMR (100 MHz, $CDCl_3$): δ 190.6, 155.8, 152.1, 148.7, 143.9, 140.6, 139.0, 131.5, 128.1, 127.4, 127.3, 126.9, 123.0, 120.5, 115.5, 68.4, 31.8, 29.5, 25.9, 22.8, 14.2.

4,4'-((4'-(bis(4-(methylthio)phenyl)amino)-[1,1'-biphenyl]-4-yl)azanediyl)dibenzaldehyde **(6d)**: In a 250 mL RBF with a magnetic stir bar, **5** (0.166 g, 0.44 mmol) and $K_3PO_4$ (0.336 g, 1.59 mmol) were dissolved in 20 mL of THF:$H_2O$ (9:1 v/v) and the mixture was sparged with $N_2$ for 30 minutes. The SMe-TPA-BPin (0.225 g, 0.48 mmol), $Pd_2(dba)_3$ catalyst (0.009 g, 0.009 mmol), and [(*t*-Bu)$_3$PH]$BF_4$ ancillary ligand (0.010 g, 0.035 mmol) were added and the mixture was heated to reflux under $N_2$ overnight. The mixture was concentrated *in vacuo*. DCM (25 mL) was added and the solution was washed with water (3 x 50 mL). The organics were collected, washed with brine, dried over $MgSO_4$ and concentrated *in vacuo*. The residue was purified by silica gel column chromatography with Hex:EtOAc (3:1 v/v) as the eluent to afford **6d** (210 mg, 0.33 mmol, 75.5%). $^1$H NMR (400 MHz, $CDCl_3$): δ = 9.90 (H*a*, s, 2H), 7.79 (H*b*, d, $^3J_{HH}$ = 8 Hz, 4H), 7.57 (H*e*, d, $^3J_{HH}$ = 8 Hz, 2H), 7.46 (H*f*, d, $^3J_{HH}$ = 8 Hz, 2H), 7.25-7.16 (H*c*,H*d*,H*h*, m, $^3J_{HH}$ = 4 Hz, 10H), 7.11 (H*g*, d, $^3J_{HH}$ = 8 Hz, 2H), 7.06 (H*i*, d, $^3J_{HH}$ = 8 Hz, 4H), 2.48 (-*SMe*, s, 6H). $^{13}C\{^1H\}$ NMR (100 MHz, $CDCl_3$): δ 190.5, 152.0, 147.2, 145.1, 144.3, 138.5, 133.8, 132.4, 131.5, 131.4, 128.6, 128.2, 127.7, 127.2, 125.1, 123.5, 123.0, 16.9.

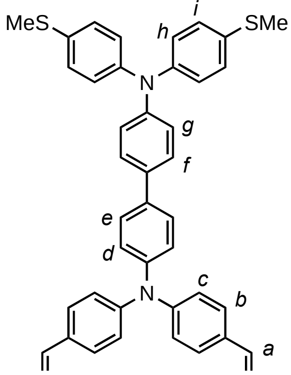

**6d**

3,3'-(((4'-(diphenylamino)-[1,1'-biphenyl]-4-yl)azanediyl)bis(4,1-phenylene))bis(2-cyanoacrylic acid) **1a:** Precursor **6a** (70 mg, 0.13 mmol) was dissolved in minimal $CHCl_3$: Hex (1:1 v/v) sparged with $N_2$ for 30 minutes. Cyanoacetic acid (0.066 g, 0.776 mmol) and piperidine (0.01 mL, 0.957 mmol) were added and the solution was heated to reflux under $N_2$ overnight (12 h). The solvent was removed, and the precipitate was dissolved in neat $CHCl_3$ and was stirred with 1.2 M HCl (10 mL). The organic phase was washed with $H_2O$ (2 x 100 mL), brine, dried over $MgSO_4$ and concentrated *in vacuo* to yield the product as a dark-orange/red solid **1a** (75 mg, 0.11 mmol, 87.6%). $^1$H NMR (400 MHz, $CDCl_3$): δ = 8.06 (H*a*, s, 2H), 7.85 (H*b*, d, $^3J_{HH}$ = 8 Hz, 4H), 7.51 (H*e*, d, $^3J_{HH}$ = 8 Hz, 2H), 7.39 (H*f*, d, $^3J_{HH}$ = 8 Hz, 2H), 7.24 (H*d*, d, $^3J_{HH}$ = 8 Hz, 4H), 7.20 (H*i*, t, $^3J_{HH}$ = 8 Hz, 4H), 7.14-7.12 (H*g*, H*c*, m, 6H), 7.06 (H*h*, d, $^3J_{HH}$ = 8 Hz, 4H), 6.97 (H*j*, t, $^3J_{HH}$ = 8 Hz, 2H). HRMS (ESI): m/z 678.22671 calculated for $C_{44}H_{30}N_4O_4$: found m/z 678.22346. *To help with 1H-NMR solubility, 2 drops of DMSO-$d^6$ was added. Poor solubility prevented the acquisition of 13C data.*

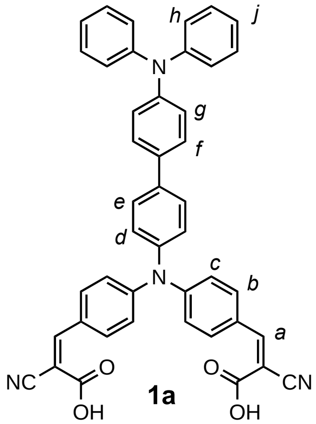

**1a**

3,3'-(((4'-(bis(4-(hexyloxy)phenyl)amino)-[1,1'-biphenyl]-4-yl)azanediyl)bis(4,1-phenylene))bis(2-cyanoacrylic acid) **1c:** Precursor **6c** (100 mg, 0.13 mmol) was dissolved in minimal $CHCl_3$: Hex (1:1 v/v) sparged with $N_2$ for 30 minutes. Cyanoacetic acid (0.066 g, 0.776 mmol) and piperidine (0.01 mL, 0.957 mmol) were added and the solution was heated to reflux under $N_2$ overnight. The liquid phase was removed, and the precipitate was dissolved in neat $CHCl_3$ and was stirred with 1.2M HCl (10 mL). The organic phase was washed with $H_2O$ (2 x 100 mL), brine, dried over $MgSO_4$ and concentrated *in vacuo* to yield the product as a dark-orange/red solid **1c** (95 mg, 0.10 mmol, 81.0%). $^1$H NMR (400 MHz, $CDCl_3$): δ = 8.06 (H*a*, s, 2H), 7.86 (H*b*, d, $^3J_{HH}$ = 8 Hz, 4H), 7.48 (H*e*, d, $^3J_{HH}$ = 8 Hz, 2H), 7.33 (H*f*, d, $^3J_{HH}$ = 8 Hz, 2H), 7.22-7.20 (H*d*, m, 2H), 7.13 (H*c*, d, $^3J_{HH}$ = 8 Hz, 4H), 7.01 (H*h*, d, $^3J_{HH}$ = 8 Hz, 4H), 6.91 (H*g*, d, $^3J_{HH}$ = 8 Hz, 2H), 6.77 (H*i*, d, $^3J_{HH}$ = 8 Hz, 4H), 3.87 (H*j*, t, $^3J_{HH}$ = 8 Hz, 4H), 1.71 (H*k*, d, 4H), 1.39 (H*l*, dt, 4H), 1.28-1.19 (H*m*,H*n*, m, 8H), 0.84 (H*o*, m, 6H). *To help with 1H-NMR solubility, 2 drops of DMSO-d$^6$ was added. Poor solubility prevented the acquisition of 13C data.*

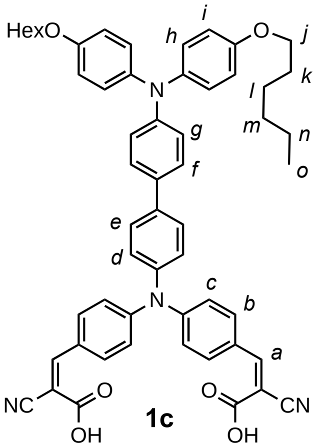


3,3'-(((4'-(bis(4-(methylthio)phenyl)amino)-[1,1'-biphenyl]-4-yl)azanediyl)bis(4,1-phenylene))bis(2-cyanoacrylic acid) **1d:** Precursor **6d** (80 mg, 0.13 mmol) was dissolved in minimal $CHCl_3$: Hex (1:1 v/v) sparged with $N_2$ for 30 minutes. Cyanoacetic acid (0.066 g, 0.776 mmol) and piperidine (0.01 mL, 0.957 mmol) were added and the solution was heated to reflux under $N_2$ overnight. The liquid phase was removed, and the precipitate was dissolved in neat $CHCl_3$ and was stirred with 1.2 M HCl (10 mL). The organic phase was washed with $H_2O$ (2 x 100 mL), brine, dried over $MgSO_4$ and concentrated *in vacuo* to yield the product as a dark-orange/red solid **1d** (80 mg, 0.10 mmol, 82.6%). $^1$H NMR (400 MHz, $CDCl_3$): δ = 8.06 (H*a*, s, 2H), 7.85 (H*b*, d, $^3J_{HH}$ = 8 Hz, 4H), 7.50 (H*e*, d, $^3J_{HH}$ = 8 Hz, 2H), 7.38 (H*f*, d, $^3J_{HH}$ = 8 Hz, 2H), 7.15 – 7.07 (H*c*, H*h*, H*d*, m, 10H), 7.03 (H*g*, d, $^3J_{HH}$ = 8 Hz, 2H), 6.98 (H*i*, d, $^3J_{HH}$ = 8 Hz, 4H), 2.41 (-*SMe*, s, 6H). HRMS (ESI): m/z 770.20215 calculated for $C_{46}H_{34}N_4O_4S_2$: Found m/z 770.20032. *To help with 1H-NMR solubility, 2 drops of DMSO-d$^6$ was added. Poor solubility prevented the acquisition of 13C data*

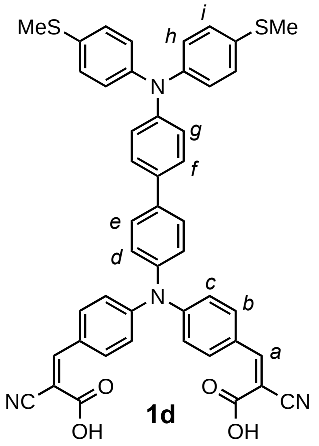

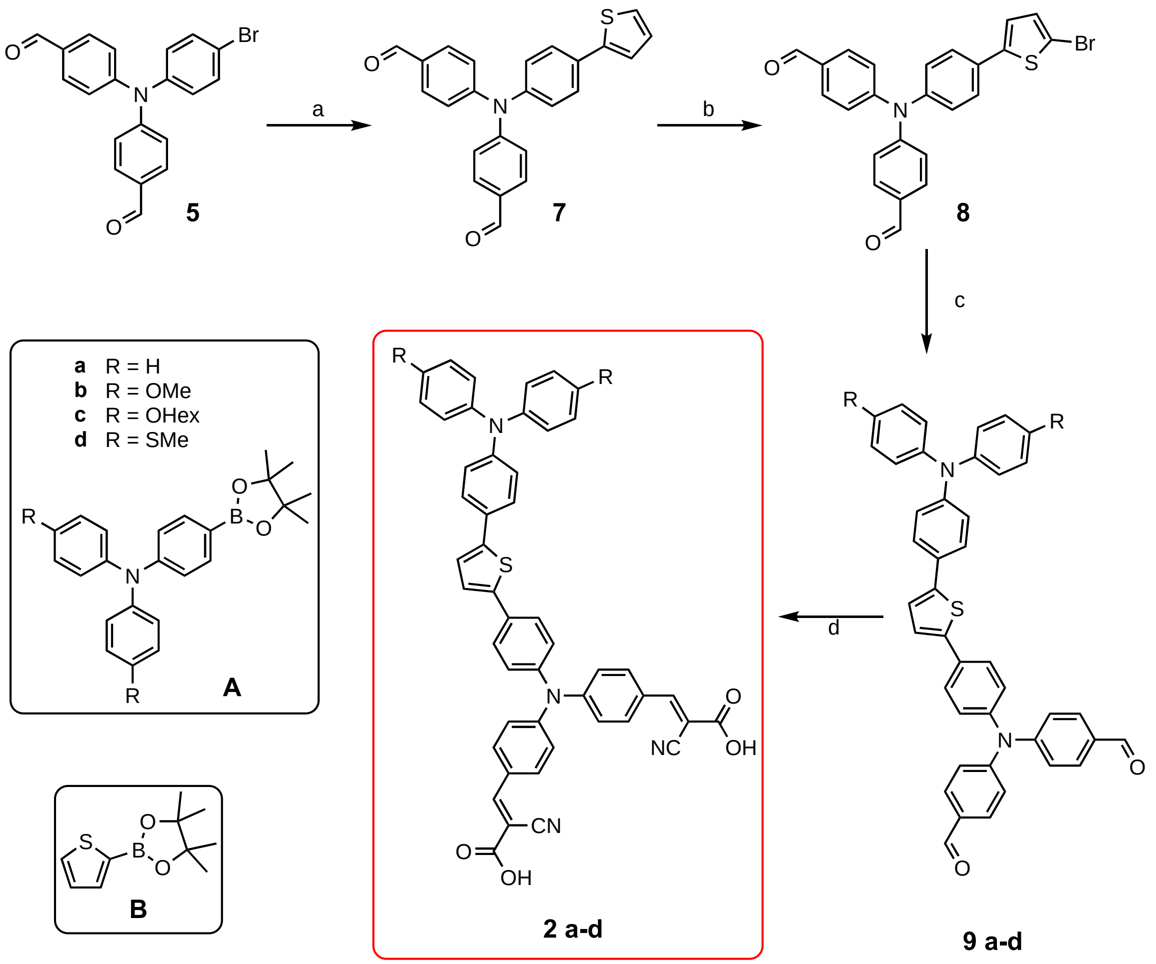


**Scheme S2.** Synthesis of aldehyde family **9 a-d** and dye family **2 a-d**. Reaction conditions: a) **B** (1.1 eq.), $K_3PO_4$ (5.1 eq.), $Pd_2(dba)_3$ (2 or 4 mol %), $[(t\text{-}Bu)_3PH]BF_4$ (1 or 2 mol %), THF:$H_2O$ (9:1 v/v), reflux 16 h. b) NBS (1.0 eq.), THF:EtOAc (2:1 v/v), 0 ℃ 5 h. c) **A** (1.1 eq.), $K_3PO_4$ (3.2 eq.), $Pd_2(dba)_3$ (2 or 4 mol %), THF:$H_2O$ (9:1 v/v), reflux 16 h. d) cyanoacetic acid (6.0 eq.), piperidine (0.100 mL), $CHCl_3$:Hex (1:1 v/v), reflux 16 h.

Molecules **Aa,**[1] **Ab,**[2] **Ac,**[3] **Ad,**[4] **B,**[6] **5,**[1] **7,**[7] **8,**[7] **9b,**[5] **2b**[5] have been previously reported

4,4'-((4-(5-(4-(diphenylamino)phenyl)thiophen-2-yl)phenyl)azanediyl)dibenzaldehyde (**9a)**: In a 250 mL RBF equipped with a magnetic stir bar, **8** (0.150 g, 0.325 mmol) and $K_3PO_4$ (0.227 g, 1.07 mmol) were dissolved in 30 mL of THF: $H_2O$ (9:1 v/v) and the mixture was sparged with $N_2$ for 30 minutes. The TPA-BPin (**A$_a$**, 0.125 g, 0.34 mmol), $Pd_2(dba)_3$ catalyst (0.007 g, 0.006 mmol), and [(*t*-Bu)$_3$PH]$BF_4$ ancillary ligand (0.007 g, 0.026 mmol) were added and the mixture was heated to reflux under $N_2$ overnight. The mixture was concentrated, extracted with DCM (100 mL) was washed with water (3 x 100 mL). The organic phases were collected, washed with brine, dried over $MgSO_4$ and concentrated *in vacuo*. The residue was purified by silica gel column chromatography with Hex:EtOAc (3:1 v/v) as the mobile phase to yield the product **9a** (120 mg, 0.19 mmol, 59.0%). $^1$H NMR (400 MHz, $CDCl_3$): δ = 9.91 (H*a*, s, 2H), 7.80 (H*b*, d, $^3J_{HH}$ = 8 Hz, 4H), 7.62 (H*e*, d, $^3J_{HH}$ = 8 Hz, 2H), 7.49 (H*h*, d, $^3J_{HH}$ = 8 Hz, 2H), 7.29–7.20 (H*d*, H*c*, H*f*, H*g*, m, 8H), 7.17 (H*i*, d, $^3J_{HH}$ = 8 Hz, 2H), 7.13 (H*j*, d, $^3J_{HH}$ = 8 Hz, 4H), 7.10-7.02 (H*k*, H*l*, m, 6H). $^{13}$C{$^1$H} NMR (100 MHz, $CDCl_3$): δ = 190.7, 151.9, 147.6, 147.5, 144.6, 144.2, 141.4, 132.4, 131.6, 131.5, 129.5, 128.2, 127.2, 127.1, 126.5, 124.7, 124.5, 123.7, 123.4, 123.1.

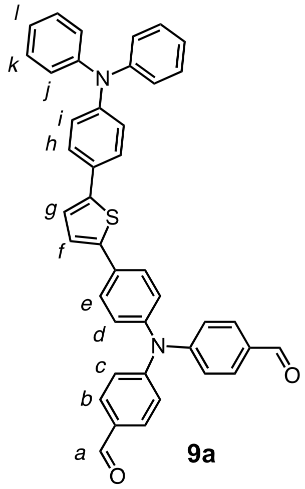

**9a**

4,4'-((4-(5-(4-(bis(4-(hexyloxy)phenyl)amino)phenyl)thiophen-2-yl)phenyl)azanediyl)dibenzaldehyde **(9c)**: In a 250 mL RBF with a magnetic stir bar, **8** (0.150 g, 0.325 mmol) and $K_3PO_4$ (0.227 g, 0.107 mmol) were dissolved in 10 mL of THF:$H_2O$ (9:1 v/v) and the mixture was sparged with $N_2$ for 30 minutes. The HexO-TPA-BPin (**A$_c$**, 0.20 g, 0.33 mmol), $Pd_2(dba)_3$ catalyst (0.007 g, 0.007 mmol), and [(*t*-Bu)$_3$PH]$BF_4$ ancillary ligand (0.007 g, 0.026 mmol) were added and the mixture was heated to reflux under $N_2$ overnight. The mixture was concentrated *in vacuo.* DCM (50 mL) was added and the solution was washed with water (3 x 100 mL). The organics were collected, washed with brine, dried over $MgSO_4$ and concentrated *in vacuo*. The residue was purified by silica gel column chromatography with Hex:EtOAc (5:1 v/v) as the mobile phase to yield the product **9c** (175 mg, 0.20 mmol, 63.1%). $^1$H NMR (400 MHz, $CDCl_3$): δ = 9.10 (H*a*, s, 2H), 7.79 (H*b*, d, $^3J_{HH}$ = 8 Hz, 4H), 7.61 (H*e*, d, $^3J_{HH}$ = 8 Hz, 2H), 7.41 (H*h*, d, $^3J_{HH}$ = 8 Hz, 2H), 7.25–7.22 (H*c*, H*d*, m, 6H), 7.17 (H*f*, d, $^3J_{HH}$ = 4 Hz, 1H), 7.15 (H*g*, d, $^3J_{HH}$ = 4 Hz, 1H), 7.06 (H*j*, d, $^3J_{HH}$ = 8 Hz, 4H), 6.92 (H*i*, d, $^3J_{HH}$ = 8 Hz, 2H), 6.83 (H*k*, d, $^3J_{HH}$ = 8 Hz, 4H), 3.94 (H*l*, t, $^3J_{HH}$ = 8 Hz, 4H), 1.78 (H*m*, dt, 4H), 1.50–1.43 (H*n*, m, 4H), 1.36–1.34 (H*o*,H*p*, m, 8H), 0.91(H*q*, t, 6H). $^{13}$C{$^1$H} NMR (100 MHz, $CDCl_3$): δ = 190.5, 155.7, 151.8, 148.6, 144.5, 144.3, 140.7, 140.4, 132.4, 131.5, 131.4, 127.1, 126.9, 126.8, 126.2, 125.9, 124.3, 122.9, 122.6, 120.3, 115.3, 68.3, 31.6, 29.3, 25.8, 22.6, 14.0.

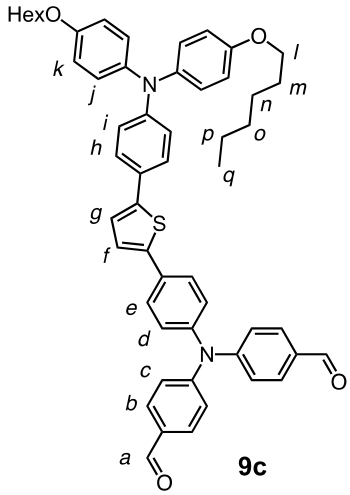

**9c**

4,4'-((4-(5-(4-(bis(4-(methylthio)phenyl)amino)phenyl)thiophen-2-yl)phenyl)azanediyl)dibenzaldehyde (**9d)**: In a 250 mL RBF with a magnetic stir bar, **8** (0.150 g, 0.325 mmol) and $K_3PO_4$ (0.227 g, 0.1.07 mmol) were dissolved in 10 mL of THF:$H_2O$ (9:1 v/v) and the mixture was sparged with $N_2$ for 30 minutes.

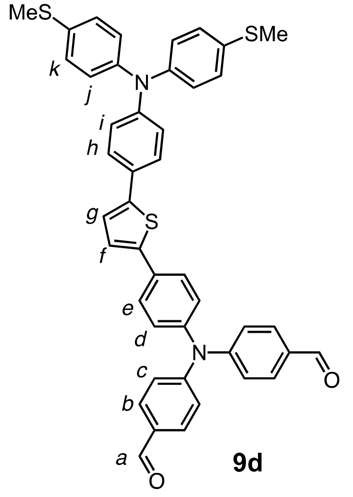


**9d**

The SMeO-TPA-BPin (**$A_d$**, 0.18 g, 0.39 mmol), $Pd_2(dba)_3$ catalyst (0.007 g, 0.007 mmol), and [(*t*-Bu)$_3$PH]$BF_4$ ancillary ligand (0.007 g, 0.026 mmol) were added and the mixture was heated to reflux under $N_2$ overnight. The mixture was concentrated *in vacuo.* DCM (50 mL) was added and the solution was washed with water (3 x 100 mL). The organics were collected, washed with brine, dried over $MgSO_4$ and concentrated *in vacuo*. The residue was purified by silica gel column chromatography with Hex:EtOAc (5:1 v/v) as the eluent to yield the product **9d** (165 mg, 0.23 mmol, 70.7%).$^1$H NMR (400 MHz, $CDCl_3$): δ = 9.91 (H*a,* s, 2H), 7.80 (H*b,* d, $^3J_{HH}$ = 8 Hz, 4H), 7.62 (H*e,* d, $^3J_{HH}$ = 8 Hz, 2H), 7.48 (H*h*, d, $^3J_{HH}$ = 8 Hz, 2H), 7.28 (H*f,* d, $^3J_{HH}$ = 4 Hz, 1H), 7.25-7.15 (H*d,* H*c,* H*g,* H*j*, m, 11H), 7.10-7.01 (H*k,* H*i,* m, 6H), 2.48 (*-SMe,* s, 6H). $^{13}$C{$^1$H} NMR (100 MHz, $CDCl_3$): δ 190.4, 151.9, 147.2, 145.0, 144.6, 144.0, 141.5, 132.5, 132.3, 131.6, 131.5, 128.6, 127.2, 127.1, 126.6, 125.1, 124.4, 123.4, 123.1, 16.7.

3,3'-(((4-(5-(4-(diphenylamino)phenyl)thiophen-2-yl)phenyl)azanediyl)bis(4,1-phenylene))bis(2-cyanoacrylic acid) (**2a)**: Precursor **9a** (100 mg, 0.16 mmol) was dissolved in minimal $CHCl_3$:Hex (1:1 v/v) and sparged with $N_2$ for 30 minutes. Cyanoacetic acid (0.082 g, 0.957 mmol) and piperidine (0.01 mL) were added and the solution was heated to reflux under $N_2$ overnight. The liquid phase was decanted, and the precipitate was dissolved in neat $CHCl_3$ and was stirred with 1.2 M HCl (20 mL). The organic phase was washed with $H_2O$ (2 x 100 mL), brine, dried over $MgSO_4$ and concentrated *in vacuo* to yield the product as a dark-orange/red solid **2a** (95 mg, 0.12 mmol, 78.3%). $^1$H NMR (400 MHz, $CDCl_3$): δ = 8.21 (H*a*, s, 2H), 7.99 (H*b,* d, $^3J_{HH}$ = 8 Hz, 4H), 7.65 (H*e*, d, $^3J_{HH}$ = 8 Hz, 2H), 7.50 (H*h*, d, $^3J_{HH}$ = 8 Hz, 2H), 7.30-7.02 (H*d,* H*c,* H*f,* H*g,* H*i,* m, $^3J_{HH}$ = 8 Hz, 10H), 7.13 (H*j,* d, $^3J_{HH}$ = 8 Hz, 4H), 7.10-7.02 (H*k,* H*l,* m, 6H). HRMS (ESI): m/z 760.21443 calculated for $C_{46}H_{32}N_4O_4S$: found m/z 760.21082. *To help with 1H-NMR solubility, 2 drops of DMSO-d$^6$ was added. Poor solubility prevented the acquisition of 13C data.*

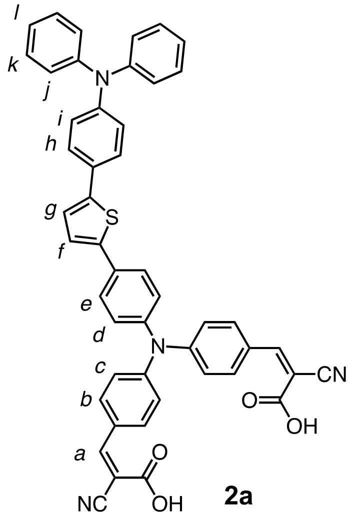


**2a**

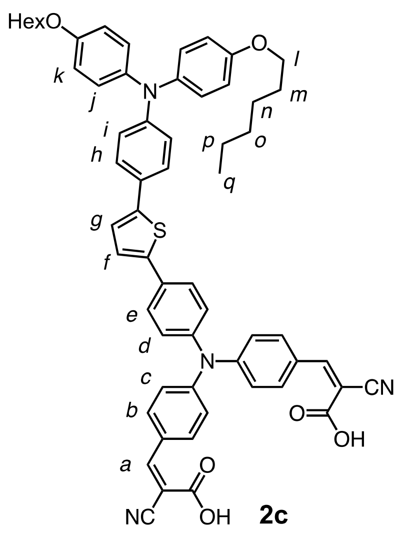


3,3'-(((4-(5-(4-(bis(4-(hexyloxy)phenyl)amino)phenyl)thiophen-2-yl)phenyl)azanediyl)bis(4,1-phenylene))bis(2-cyanoacrylic acid) (**2c**): Precursor **9c** (135 mg, 0.16 mmol) was dissolved in minimal $CHCl_3$:Hex (1:1 v/v) sparged with $N_2$ for 30 minutes. Cyanoacetic acid (0.082 g, 0.957 mmol) and piperidine (0.01 mL) were added and the solution was heated to reflux under $N_2$ overnight. The liquid phase was removed and the precipitate was dissolved in neat $CHCl_3$ and was stirred with 1.2M HCl (10 mL). The organic phase was washed with $H_2O$ (2 x 100 mL), brine, dried over $MgSO_4$ and concentrated *in vacuo* to yield the product as a dark-orange/red solid **2c** (112 mg, 0.11 mmol, 71.7%). $^1$H NMR (400 MHz, $CDCl_3$): δ = 8.14 (H*a,* s, 2H), 7.87 (H*b*, br m, 4H), 7.56 (H*e*, br m, 2H), 7.40 (H*h*, H*d*, br m, 4H), 7.30–7.00 (H*c,* H*f*, H*g*, H*i*, m, 8H), 6.91 (H*j*, m, 4H), 6.82 (H*k*, m, 4H), 3.93 (H*l*, m, 4H), 1.76 (*m*, m, 4H), 1.45 (*n*, m, 4H), 1.30–1.28 (H*o, Hp,* m, 8H), 0.91 (H*q*, m, 6H). *To help with 1H-NMR solubility, 2 drops of DMSO-d$^6$ was added. Poor solubility prevented the acquisition of 13C data.*

(2*Z*,2'Z)-3,3'-(((4-(5-(4-(bis(4-(methylthio)phenyl)amino)phenyl)thiophen-2-yl)phenyl)azanediyl)bis(4,1-phenylene))bis(2-cyanoacrylic acid) (**2d**)**:** Precursor **9d** (115 mg, 0.16 mmol) was dissolved in minimal $CHCl_3$:Hex (1:1 v/v) sparged with $N_2$ for 30 minutes. Cyanoacetic acid (0.082 g, 0.957 mmol) and piperidine (0.01 mL) were added and the solution was heated to reflux under $N_2$ overnight. The liquid phase was removed and the precipitate was dissolved in neat $CHCl_3$ and was stirred with 1.2M HCl (10 mL). The organic phase was washed with $H_2O$ (2 x 100 mL), brine, dried over $MgSO_4$ and concentrated *in vacuo* to yield the product **2d** as a dark-orange/red solid (110 mg, 0.13 mmol, 80.6%). $^1$H NMR (400 MHz, $CDCl_3$): δ = 8.22 (H*a*, s, 2H), 7.99 (H*b*, d, $^3J_{HH}$ = 8 Hz, 4H), 7.65 (H*e*, d, $^3J_{HH}$ = 8 Hz, 2H), 7.49 (H*h*, d, $^3J_{HH}$ = 8 Hz, 2H), 7.30-7.15 (H*f*, H*d*, H*c*, H*g*, H*j*, m, 12H), 7.10-6.95 (H*k*, H*i*, m, 6H), 2.48 (-*SMe*, s, 6H). HRMS (ESI-negative mode): m/z 851.18259 calculated for $C_{50}H_{35}N_4O_4S_3^-$: Found m/z 851.18658. *To help with 1H-NMR solubility, 2 drops of DMSO-d$^6$ was added. Poor solubility prevented the acquisition of 13C data.*

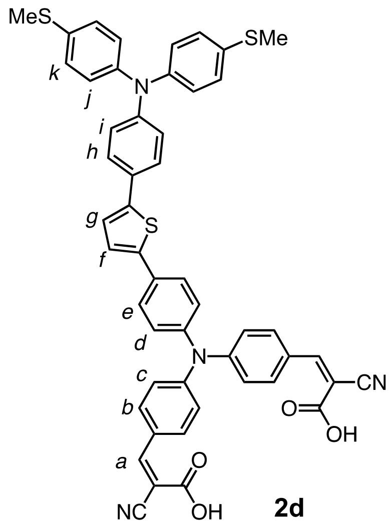

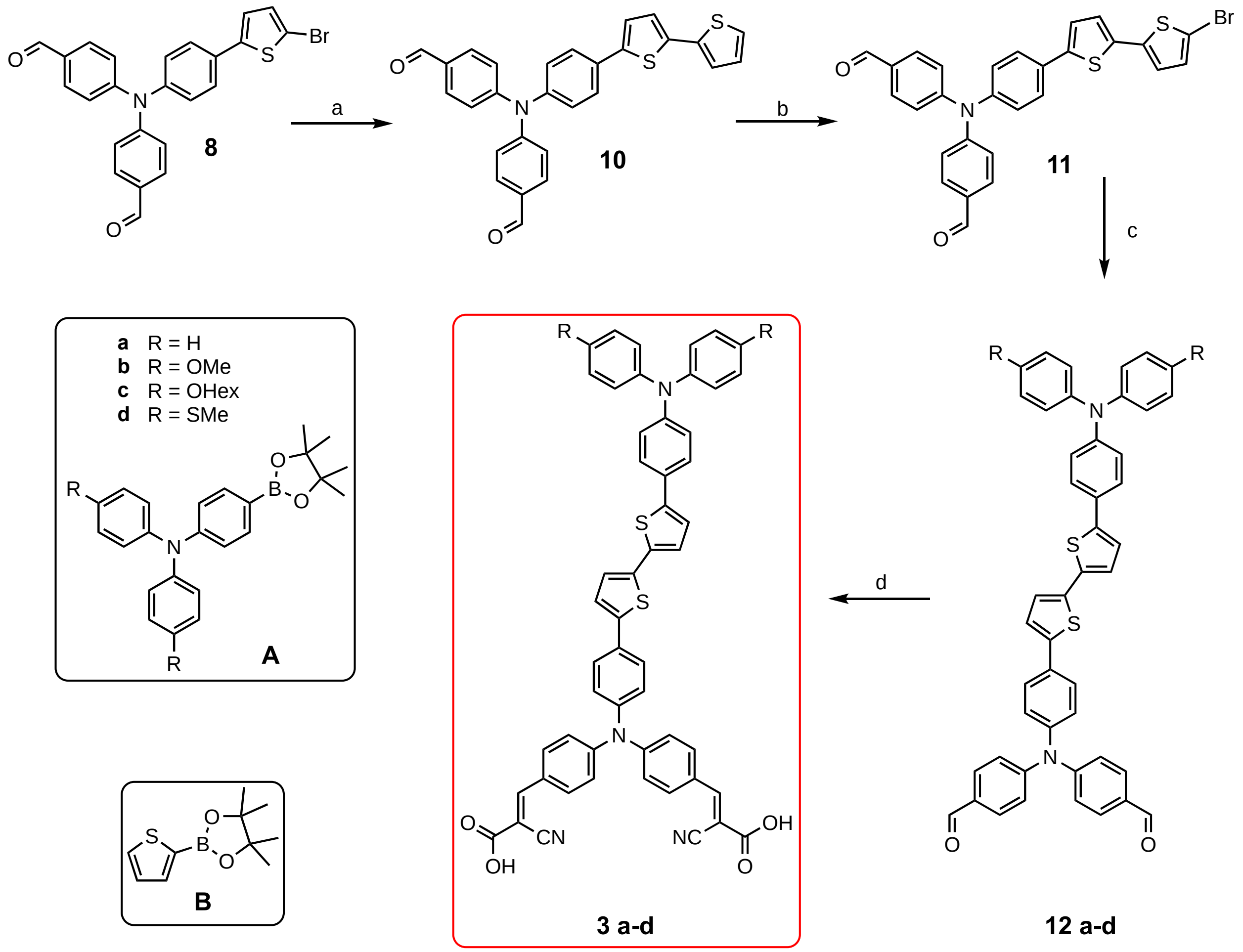


**Scheme S3.** Synthesis of aldehyde family **12 a-d** and dye family **1 a-d**. Reaction conditions: a) **B** (1.1 eq.), $K_3PO_4$ (5.1 eq.), $Pd_2(dba)_3$ (2 or 4 mol %), [(t-Bu)$_3$PH]$BF_4$ (1 or 2 mol %), THF:$H_2O$ (9:1 v/v), reflux 16 h. b) NBS (1.0 eq.), THF:EtOAc (2:1 v/v), 0 ℃ 5 h. c) **B** (1.1 eq.), $K_3PO_4$ (3.3 eq.), $PdCl_2(PPh_3)_2$ (10 mol %), THF:$H_2O$ (9:1 v/v), reflux 16 h. d) NBS (1.0 eq.), THF:EtOAc (2:1 v/v), 0 ℃ 5 h. e) **A** (1.1 eq.), $K_3PO_4$ (3.2 eq.), $Pd_2(dba)_3$ (2 or 4 mol %), THF:$H_2O$ (9:1 v/v), reflux 16 h. f) cyanoacetic acid (6.0 eq.), piperidine (0.100 mL), $CHCl_3$:Hex (1:1 v/v), reflux 16 h.

Molecules **Aa,**[1] **Ab,**[2] **Ac,** [3] **Ad,**[4] **B,**[6] **8,**[7] have been previously reported

4,4'-((4-([2,2'-bithiophen]-5-yl)phenyl)azanediyl)dibenzaldehyde (**10**)**:** In a 250 mL RBF with a magnetic stir bar, **8** (0.500 g, 1.08 mmol) and $K_3PO_4$ (0.758 g, 3.57 mmol) were dissolved in 20 mL THF:$H_2O$ (9:1 v/v) and the mixture was sparged with $N_2$ for 30 minutes. The $Pd_2(dba)_3$ catalyst (0.045 g, 0.043 mmol), [(*t*-Bu)$_3$PH]$BF_4$ ancillary ligand (0.024 g, 0.087 mmol) and thiophene boronic ester (**B**, 0.273 g, 1.30 mmol) were added and the mixture was heated to reflux under $N_2$ overnight. The solution was concentrated *in vacuo*, re-dissolved in DCM (50 mL), washed with $H_2O$ (2 x 100 mL) and brine (50 mL). The organics collected and dried over $MgSO_4$ and concentrated *in vacuo*. The product was purified by silica gel chromatography in 5:1 Hex:EtOAc to afford a yellow solid as **10** (450 mg, 0.97 mmol, 89.4%). $^1$H NMR (400 MHz, $CDCl_3$): δ = 9.91 (H*a*, s, 2H), 7.80 (H*b*, d, $^3J_{HH}$ = 8 Hz, 4H), 7.60 (H*e*, d, $^3J_{HH}$ = 8 Hz, 2H), 7.24–7.21 (H*d*, H*c*, H*f*, H*g*, H*g*, H*j*, m, 10H), 7.05 (H*i*, dd, 1H). $^{13}$C{$^1$H} NMR (100 MHz, $CDCl_3$): δ 190.5, 151.8, 144.78, 141.9, 137.3, 137.2, 131.9, 131.6, 131.45, 128.0, 127.2, 127.1, 124.8, 124.7, 124.1, 123.9, 123.1.

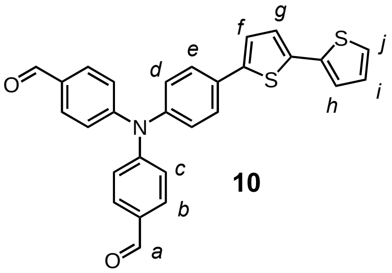

**10**

4,4'-((4-(5'-bromo-[2,2'-bithiophen]-5-yl)phenyl)azanediyl)dibenzaldehyde (**11**): In a 250 mL RBF with a magnetic stir bar, **10** (0.250 g, 0.537 mmol) was dissolved in 45 mL of 2:1 THF:EtOAc and cooled in an ice bath. The solution was sparged with $N_2$ vigorously for 30 minutes. The flask was covered with aluminium foil and the NBS (0.096 g, 0.537 mmol) was added. The mixture was capped with a rubber septum and stirred at 0 °C for 1 h, brought up to room temperature and stirred for another 5 h. The solvents were removed *in vacuo* and the product was purified via a short silica gel plug with 4:1 Hex:EtOAc (v/v) as the eluent to yield **11** (275 mg, 0.51 mmol, 94.1%). $^1$H NMR (400 MHz, $CDCl_3$): δ = 9.91 (H*a*, s, 2H), 7.80 (H*b*, d, $^3J_{HH}$ = 8 Hz, 4H), 7.58 (H*e*, d, $^3J_{HH}$ = 8 Hz, 2H), 7.23 (H*c*, d, $^3J_{HH}$ = 8 Hz, 4H), 7.20 (H*f*, d, $^3J_{HH}$ = 4 Hz, 1H), 7.17 (H*d*, d, $^3J_{HH}$ = 8 Hz, 2H), 7.10 (H*g*, d, $^3J_{HH}$ = 4 Hz, 1H), 6.99 (H*h*, d, $^3J_{HH}$ = 4 Hz, 1H), 6.95 (H*i*, d, $^3J_{HH}$ = 4 Hz, 1H). $^{13}$C{$^1$H} NMR (100 MHz, $CDCl_3$): δ 190.5, 151.8, 145.0, 142.4, 138.8, 136.1, 131.7, 131.7, 131.5, 130.8, 127.2, 127.1, 125.0, 124.1, 123.9, 123.2.

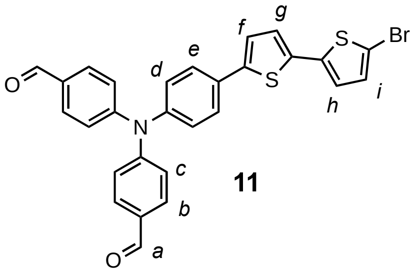

**11**

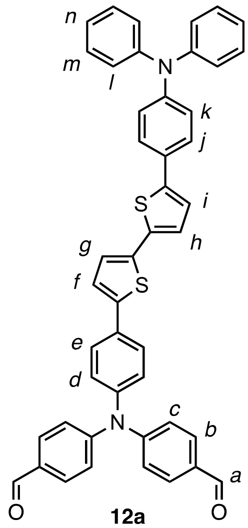
12a

4,4'-((4-(5'-(4-(diphenylamino)phenyl)-[2,2'-bithiophen]-5-yl)phenyl)azanediyl)dibenzaldehyde (**12a):** In a 100 mL RBF with a magnetic stir bar, **11** (0.150 g, 0.275 mmol) and $K_3PO_4$ (0.193 g, 0.909 mmol) were dissolved in 50 mL of THF: $H_2O$ (9:1 v/v) and the mixture was sparged with $N_2$ for 30 minutes. The TPA-BPin (**A$_a$**, 0.106 g, 0.286 mmol), $Pd_2(dba)_3$ catalyst (0.006 g, 0.006 mmol), and [(*t*-Bu)$_3$PH]$BF_4$ ancillary ligand (0.003 g, 0.009 mmol) were added and the mixture was heated to reflux under nitrogen overnight. The mixture was then concentrated *in vacuo.* DCM (50 mL) was added and the solution was washed with water (3 x 100 mL). The organics were collected, washed with brine, dried over $MgSO_4$ and concentrated *in vacuo*. The residue was purified by silica gel column chromatography with Hex:EtOAc (5:1 v/v) to yield **12a** as a yellow solid (175 mg, 0.25 mmol, 89.7%). $^1$H NMR (400 MHz, $CDCl_3$): δ = 9.91 (H*a,* s, 2H), 7.80 (H*b,* d, $^3J_{HH}$ = 8 Hz, 4H), 7.60 (H*e,* d, $^3J_{HH}$ = 8 Hz, 2H), 7.46 (H*j,* d, $^3J_{HH}$ = 8 Hz, 2H), 7.30–7.22 (H*f,* H*c,* H*d,* H*g,* H*h,* m, 9H), 7.20–7.10 (H*i,* H*l* , H*m,* m, 9H), 7.10–7.00 (H*k,* H*n,* m, 4H). $^{13}$C{$^1$H} NMR (100 MHz, $CDCl_3$): δ 190.6, 151.8, 147.5, 144.7, 143.5, 131.6, 131.5, 129.4, 127.1, 126.5, 124.7, 124.1, 123.6, 123.3, 123.1; *Poor solubility prevented the acquisition of complete 13C data.*

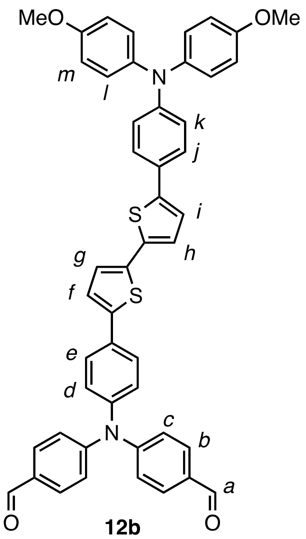
12b

4,4'-((4-(5'-(4-(bis(4-methoxyphenyl)amino)phenyl)-[2,2'-bithiophen]-5-yl)phenyl)azanediyl)dibenzaldehyde (**12b)**: In a 100 mL RBF with a magnetic stir bar, **11** (0.150 g, 0.275 mmol) and $K_3PO_4$ (0.193 g, 0.909 mmol) were dissolved in 50 mL of THF: $H_2O$ (9:1 v/v) and the mixture was sparged with $N_2$ for 30 minutes. The OMe-TPA-BPin (**A$_b$**, 0.140 g, 0.325 mmol), $Pd_2(dba)_3$ catalyst (0.011 g, 0.011 mmol), and [(*t*-Bu)$_3$PH]$BF_4$ ancillary ligand (0.006 g, 0.022 mmol) were added and the mixture was heated to reflux under nitrogen overnight. The mixture was then concentrated *in vacuo.* DCM (50 mL) was added and the solution was washed with water (3 x 100 mL). The organics were collected, washed with brine, dried over $MgSO_4$ and concentrated *in vacuo*. The residue was purified by silica gel column chromatography with Hex:EtOAc (5:1 v/v) to yield **12b** as a yellow solid (170 mg, 0.22 mmol, 80.3%). $^1$H NMR (400 MHz, $CDCl_3$): δ = 9.91 (H*a*, s, 2H), 7.80 (H*b,* d, $^3J_{HH}$ = 8 Hz, 4H), 7.59 (H*e,* d, $^3J_{HH}$ = 8 Hz, 2H), 7.39 (H*j,* d, $^3J_{HH}$ = 8 Hz, 2H), 7.24–7.07(H*f,* H*c,* H*d,* H*g,* H*h,* H*i,* H*l,* m, 14H), 6.92 (H*k,* d, $^3J_{HH}$ = 8 Hz, 2H), 6.85 (H*m,* d, $^3J_{HH}$ = 8 Hz, 4H), 3.81 (-*OMe*, s, 6H). $^{13}$C{$^1$H} NMR: *Poor solubility or aggregation prevented the acquisition of complete 13C data.*

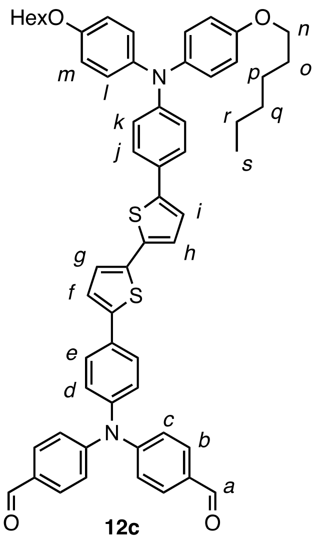

12c

((4-(5'-(4-(bis(4-formylphenyl)amino)phenyl)-[2,2'-bithiophen]-5-yl)phenyl)azanediyl)bis(4,1-phenylene) dihexanoate (**12c)**: In a 100 mL RBF with a magnetic stir bar, **11** (0.150 g, 0.275 mmol) and $K_3PO_4$ (0.193 g, 0.909 mmol) were dissolved in 50 mL of THF: $H_2O$ (9:1 v/v) and the mixture was sparged with $N_2$ for 30 minutes. The OHex-TPA-BPin ($\mathbf{A_c}$, 0.182 g, 0.303 mmol), $Pd_2(dba)_3$ catalyst (0.006 g, 0.006 mmol), and $[(t\text{-Bu})_3PH]BF_4$ ancillary ligand (0.003 g, 0.009 mmol) were added and the mixture was heated to reflux under nitrogen overnight. The mixture was then concentrated *in vacuo.* DCM (50 mL) was added and the solution was washed with water (3 x 100 mL). The organics were collected, washed with brine, dried over $MgSO_4$ and concentrated *in vacuo*. The residue was purified by silica gel column chromatography with Hex:EtOAc (5:1 v/v) to yield **12c** as a yellow oil that solidified upon standing (200 mg, 0.21 mmol, 77.5%). $^1$H NMR (400 MHz, $CDCl_3$): δ = 9.91 (H*a*, s, 2H), 7.80 (H*b*, d, $^3J_{HH}$ = 8 Hz, 4H), 7.60 (H*e*, d, $^3J_{HH}$ = 8 Hz, 2H), 7.39 (H*j*, d, $^3J_{HH}$ = 8 Hz, 2H), 7.25–7.21 (H*f*, H*c*, m, 5H), 7.17 (H*d*, d, $^3J_{HH}$ = 8 Hz, 2H), 7.13 (H*g*, H*h*, m, 2H), 7.09 (H*i*, d, $^3J_{HH}$ = 4 Hz, 2H), 7.06 (H*l*, d, $^3J_{HH}$ = 8 Hz, 4H), 6.91 (H*k*, d, $^3J_{HH}$ = 8 Hz, 2H), 6.84 (H*m*, d, $^3J_{HH}$ = 8 Hz, 4H), 3.94 (H*n*, d, $^3J_{HH}$ = 8 Hz, 4H), 1.78 (H*o*, dt, 4H), 1.46 (H*p*, dt, 4H), 1.36–1.34 (H*q*, H*r*, m, 8H), 0.92 (H*s*, t, 6H). $^{13}$C{$^1$H} NMR (100 MHz, $CDCl_3$): δ = 190.6, 155.8, 151.8, 148.6, 144.7, 144.0, 141.4, 140.4, 137.6, 135.0, 132.0, 131.6, 131.5, 127.2, 127.1, 126.9, 126.3, 125.8, 124.8, 124.2, 124.1, 123.1, 122.4, 120.3, 115.4, 68.4, 31.7, 29.4, 25.9, 22.7, 14.1.

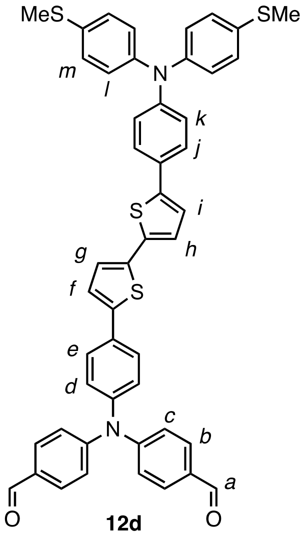

12d

4,4'-((4-(5'-(4-(bis(4-(methylthio)phenyl)amino)phenyl)-[2,2'-bithiophen]-5-yl)phenyl)azanediyl)dibenzaldehyde (**12d)**: In a 100 mL RBF with a magnetic stir bar, **11** (0.150 g, 0.275 mmol) and $K_3PO_4$ (0.193 g, 0.909 mmol) were dissolved in 50 mL of THF: $H_2O$ (9:1 v/v) and the mixture was sparged with $N_2$ for 30 minutes. The SMe-TPA-BPin ($\mathbf{A_d}$, 0.170 g, 0.370 mmol), $Pd_2(dba)_3$ catalyst (0.016 g, 0.015 mmol), and $[(t\text{-Bu})_3PH]BF_4$ ancillary ligand (0.009 g, 0.031 mmol) were added and the mixture was heated to reflux under nitrogen overnight. The mixture was then concentrated *in vacuo.* DCM (50 mL) was added and the solution was washed with water (3 x 100 mL). The organics were collected, washed with brine, dried over $MgSO_4$ and concentrated *in vacuo.* The residue was purified by silica gel column chromatography with Hex:EtOAc (5:1 v/v) to yield **12d** as a yellow solid (180 mg, 0.22 mmol, 81.7%). $^1$H NMR (400 MHz, $CDCl_3$): δ = 9.91 (H*a*, s, 2H), 7.80 (H*b*, d, $^3J_{HH}$ = 8 Hz, 4H), 7.60 (H*e*, d, $^3J_{HH}$ = 8 Hz, 2H), 7.45 (H*j*, d, $^3J_{HH}$ = 8 Hz, 2H), 7.26–7.13 (H*f*, H*c*, H*d*, H*g*, H*h*, H*i*, H*l*, m, 14H), 7.04 (H*m*, H*k*, d, $^3J_{HH}$ = 8 Hz, 6H), 2.48 (-*SMe*, s, 6H). $^{13}$C{$^1$H} NMR (100 MHz, $CDCl_3$): δ = 190.6, 151.8, 147.2, 144.9, 144.8, 143.4, 141.7, 137.4, 135.7, 132.5, 131.9, 131.7, 131.5, 128.6, 128.1, 127.1, 126.5, 125.1, 124.8, 124.5, 124.2, 123.4, 123.1, 123.0, 16.8.

3,3'-(((4-(5'-(4-(diphenylamino)phenyl)-[2,2'-bithiophen]-5-yl)phenyl)azanediyl)bis(4,1-phenylene))bis(2-cyanoacrylic acid) (**3a)**: Precursor **12a** (0.100 g, 0.141 mmol) was dissolved in minimal $CHCl_3$:Hex (1:1 v/v) and was sparged with $N_2$ for 30 minutes. Cyanoacetic acid (0.072 g, 0.846 mmol) and piperidine (0.1 mL) were added and the solution was heated to reflux under $N_2$ overnight. The liquid phase was decanted. The precipitate was dissolved in $CHCl_3$ and was washed with 1.2 M HCl (2 x 50 mL). The organic phase was washed with brine, dried over $MgSO_4$ and concentrated *in vacuo* to yield **3a** as a dark red solid (105 mg, 0.125 mmol, 88.2%). $^1$H NMR (400 MHz, $CDCl_3$): δ = 7.82 (H*a*, s, 2H), 7.62 (H*b*, d, $^3J_{HH}$ = 8 Hz, 4H), 7.31-7.25 (H*e*,H*j*, d, $^3J_{HH}$ = 8 Hz, 4H), 6.98-6.68 (H*f*, H*c*, H*d*, H*g*, H*h*, H*i*, H*l*, H*k*, H*m*, H*n*, m, 23H). *To help with 1H-NMR solubility, 2 drops of DMSO-d$^6$ was added. Poor solubility prevented the acquisition of 13C data.*

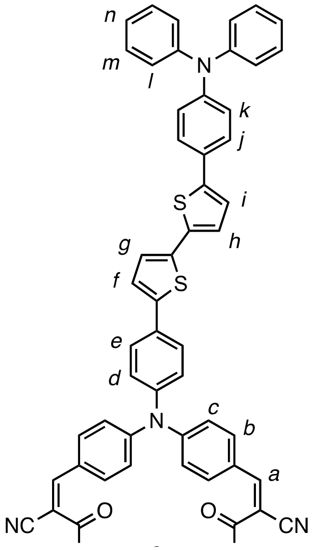

**3a**

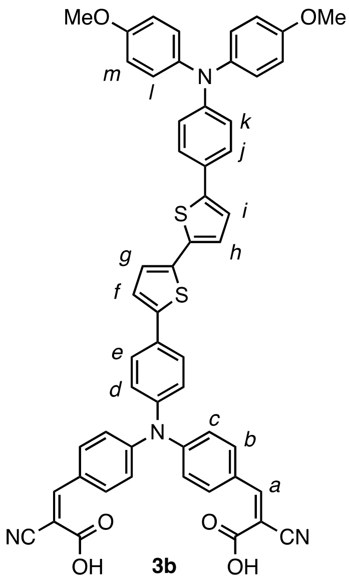

**3b**

3,3'-(((4-(5'-(4-(bis(4-methoxyphenyl)amino)phenyl)-[2,2'-bithiophen]-5-yl)phenyl)azanediyl)bis(4,1-phenylene))bis(2-cyanoacrylic acid) (**3b)**: Precursor **12b** (0.110 g, 0.141 mmol) was dissolved in minimal $CHCl_3$:Hex (1:1 v/v) and was sparged with $N_2$ for 30 minutes. Cyanoacetic acid (0.072 g, 0.846 mmol) and piperidine (0.1 mL) were added and the solution was heated to reflux under $N_2$ overnight. The liquid phase was decanted. The precipitate was dissolved in $CHCl_3$ and was washed with 1.2 M HCl (2 x 50 mL). The organic phase was washed with brine, dried over $MgSO_4$ and concentrated *in vacuo* to yield **3b** as a dark-orange/red solid (110 mg, 0.122 mmol, 85.1%). $^1$H NMR (400 MHz, $CDCl_3$): δ = 7.90 (H*a*, s, 2H), 7.67 (H*b*, br m, 4H), 7.40-6.50 (*all other protons, m),* 3.57 (*-OMe,* s, 6H). HRMS (ESI-negative mode): m/z 901.21600 calculated for $C_{54}H_{37}N_4O_6S_2^-$: found m/z 901.22018. *To help with 1H-NMR solubility, 2 drops of DMSO-d$^6$ was added.Still the 1HNMR data is incomplete, but a lack of aldehyde signal, physicochemical data and similar clustering of signal suggest the product is present without aldehyde contamination. Poor solubility/aggregation prevented the acquisition of 13C data.*

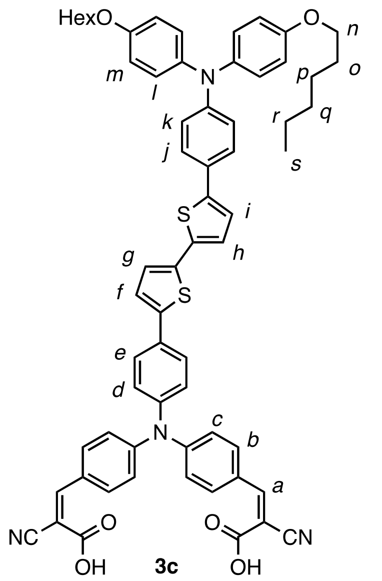


**3c**

3,3'-(((4-(5'-(4-(bis(4-(hexyloxy)phenyl)amino)phenyl)-[2,2'-bithiophen]-5-yl)phenyl)azanediyl)bis(4,1-phenylene))bis(2-cyanoacrylic acid) (**3c)**: Precursor **12c** (0.130 g, 0.141 mmol) was dissolved in minimal $CHCl_3$:Hex (1:1 v/v) and was sparged with $N_2$ for 30 minutes. Cyanoacetic acid (0.072 g, 0.846 mmol) and piperidine (0.1 mL) were added and the solution was heated to reflux under $N_2$ overnight. The liquid phase was decanted. The precipitate was dissolved in $CHCl_3$ and was washed with 1.2 M HCl (2 x 50 mL). The organic phase was washed with brine, dried over $MgSO_4$ and concentrated *in vacuo* to yield **3c** as a dark orange solid (120 mg, 0.112 mmol, 80.7%) $^1$H NMR (400 MHz, $CDCl_3$): $\delta$ = 8.15 (H*a*, s, 2H), 7.91 (H*b*, d, $^3J_{HH}$ = 8 Hz, 4H), 7.58 (H*e*, H*j*, m, 2H), 7.92 (*d*, d,$^3J_{HH}$ = 8 Hz, 2H), 7.41–6.88 (all other protons), 6.83 (H*l*, H*m*, m, 8H), 3.93 (H*n*, m, 4H), 2.86 (H*o*, m, 4H), 1.77 (H*p*, m, 4H), 1.45 (H*q*, m, 4H), 1.34 (H*r*, m, 4H), 0.91 (H*s*, m, 6H). *To help with 1H-NMR solubility, 2 drops of DMSO-d$^6$ was added. Poor solubility prevented the acquisition of better quality 1H and 13C data.*

3,3'-(((4-(5'-(4-(bis(4-(methylthio)phenyl)amino)phenyl)-[2,2'-bithiophen]-5-yl)phenyl)azanediyl)bis(4,1-phenylene))bis(2-cyanoacrylic acid) (**3d)**: Precursor **12d** (0.115 g, 0.141 mmol) was dissolved in minimal $CHCl_3$:Hex (1:1 v/v) and was sparged with $N_2$ for 30 minutes. Cyanoacetic acid (0.072 g, 0.846 mmol) and piperidine (0.1 mL) were added and the solution was heated to reflux under $N_2$ overnight. The liquid phase was decanted, and the precipitate was dissolved in $CHCl_3$ and was washed with 1.2 M HCl (2 x 50 mL). The organic phase was washed with brine, dried over $MgSO_4$ and concentrated *in vacuo* to yield **3d** as a dark red solid (110 mg, 0.118 mmol, 81.8%). $^1$H NMR (400 MHz, $CDCl_3$): $\delta$ = 8.10 (H*a*, s, 2H), 7.89 (H*b*, d, $^3J_{HH}$ = 8 Hz, 4H), 7.56 (H*e*, d, $^3J_{HH}$ = 8 Hz, 2H), 7.42 (H*j*, d, $^3J_{HH}$ = 8 Hz, 2H), 7.30–7.05 (H*f*, H*c*, H*d*, H*g*, H*h*, H*i*, m, 10H), 7.01 (H*l*, H*m*, H*k*, m, 10H), 2.44 (-*SMe*, s, 6H). *To help with 1H-NMR solubility, 2 drops of DMSO-d$^6$ was added. Poor solubility prevented the acquisition of 13C data.*

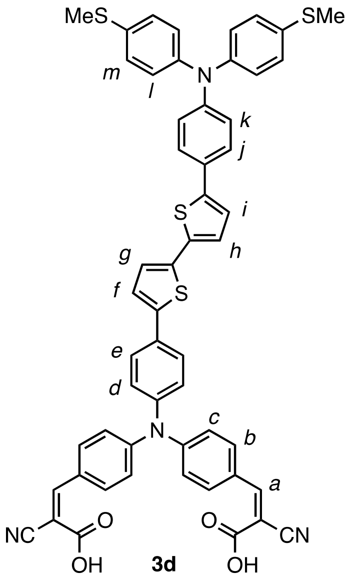


**3d**

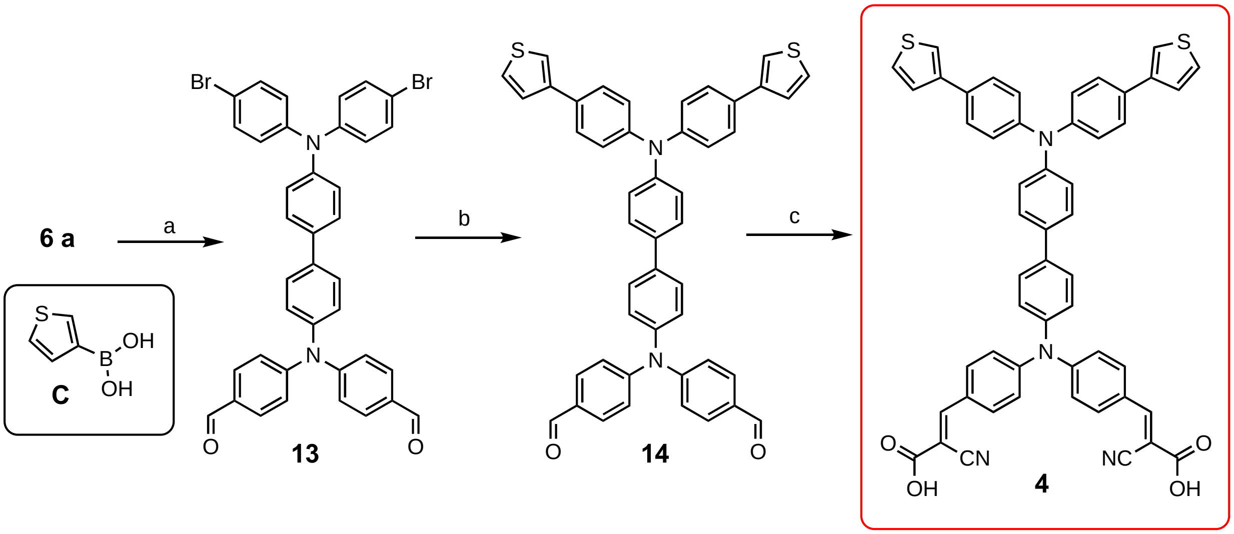


**Scheme S4.** Synthesis of dye **4**. Reaction conditions: a) NBS (2.1 eq.), THF:EtOAc (1:1 v/v), 22 ℃, 8 h. c) **C** (2.5 eq.), $K_3PO_4$ (3.5 eq.), $Pd(PPh_3)_4$ (10 mol %), dioxane:$H_2O$ (4:1 v/v), reflux 16 h. c) cyanoacetic acid (5.5 eq.), piperidine (0.100 mL), MeCN, reflux 16 h.

Molecules **6a**[1] has been previously reported.

4,4'-((4'-(bis(4-bromophenyl)amino)-[1,1'-biphenyl]-4-yl)azanediyl)dibenzaldehyde (**13**)**:** To a sparged (15 min with Ar) solution of **6a** (1.11 g, 2.04 mmol) in THF:EtOAc (1:1, 50 mL) was added NBS (763 mg, 4.28 mmol). The RBF reaction vessel was wrapped in aluminium foil, and stirred at room temperature overnight to yield an orange solution. The solvent was removed in vacuo and the crude mixture was subjected to column chromatography ($SiO_2$, DCM), to afford **13** a yellow oil ($R_f$ = 0.40, 1.30 g, 1.85 mmol, 90.7%), that solidified as a foam when the solvent was removed. $^1$H NMR (400 MHz, $CDCl_3$): δ = 9.91 (H*a*, s, 2H), 7.80 (H*b*, d, $^3J_{HH}$ = 8 Hz, 4H), 7.58 (H*e*, d, $^3J_{HH}$ = 8 Hz, 2H), 7.49 (H*f*, d, $^3J_{HH}$ = 8 Hz, 2H), 7.34 (H*c*, d, $^3J_{HH}$ = 8 Hz, 4H), 7.23 (H*d*, d, $^3J_{HH}$ = 8 Hz, 2H), 7.12 (H*g*, d, $^3J_{HH}$ = 8 Hz, 2H), 6.98 (H*h*, d, $^3J_{HH}$ = 8 Hz, 4H), 6.87 (H*i*, d, $^3J_{HH}$ = 8 Hz, 4H). $^{13}$C NMR (100 MHz, $CDCl_3$) δ 190.1, 151.6, 147.4, 142.0, 131.5, 131.4, 130.8, 129.4, 128.6, 128.2, 127.7, 127.4, 127.1, 126.9, 124.6, 122.9, 119.5. HRMS (MALDI): m/z 700.03561 calculated for $C_{38}H_{26}Br_2N_2O_2$: m/z 700.03610.

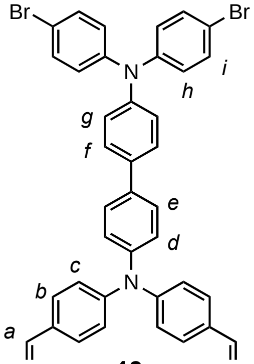

**13**

4,4'-((4'-(bis(4-(thiophen-3-yl)phenyl)amino)-[1,1'-biphenyl]-4-yl)azanediyl)dibenzaldehyde (**14**)**:** To a sparged (30 min with Ar) solution of 3-thienyl boronic acid, **C**, (550 mg, 4.27 mmol) and **13** (1.00 g, 1.42 mmol) in dioxane: water (4:1, 50 mL) was added tripotassium phosphate (1.50 g, 7.10 mmol) and finally $Pd(PPh_3)_4$ (150 mg, 0.14 mmol). The reaction mixture was then stirred and heated (105 °C) overnight (12 hrs) under Ar. After extracting with DCM and washing with water, the organic fractions were dried over sodium sulphate, filtered and rotovapped to dryness. The crude mixture was then subjected to gradient column chromatography ($SiO_2$) initially eluting with DCM and shifting to DCM:EtOAc (48:2). The product ($R_f$ = 0.5 in DCM:EtOAc, 95:5) was isolated as a yellow solid (720 mg, 1.01 mmol, 71.5%).$^1$H NMR (400 MHz, $CDCl_3$): δ = 9.91 (H*a*, s, 2H), 7.79 (H*b*, d, $^3J_{HH}$ = 8 Hz, 4H), 7.59 (H*e*, d, $^3J_{HH}$ = 8 Hz, 2H), 7.50 (H*f*, d, $^3J_{HH}$ = 8 Hz, 2H), 7.38 (H*c*, d, $^3J_{HH}$ = 8 Hz, 4H), 7.33 (H*l*, s, 2H), 7.26 – 7.20 (H*d*, H*g*, H*j*, H*k*, m, 8H), 7.19 (H*h*, d, $^3J_{HH}$ = 8 Hz, 4H), 6.99 (H*i*, d, $^3J_{HH}$ = 8 Hz, 4H). $^{13}$C NMR (100 MHz, $CDCl_3$): δ 190.5, 151.9, 146.2, 141.8, 131.5, 131.4, 130.8, 129.4, 128.6, 128.2, 127.7, 127.4, 127.1, 126.9, 126.1, 124.6, 123.0, 122.9, 122.1, 119.5, 119.0. HRMS (MALDI): m/z 708.19018 calculated for $C_{46}H_{32}N_2O_2S_2$: m/z 708.19052.

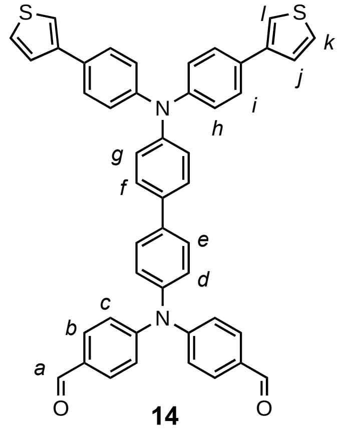

**14**

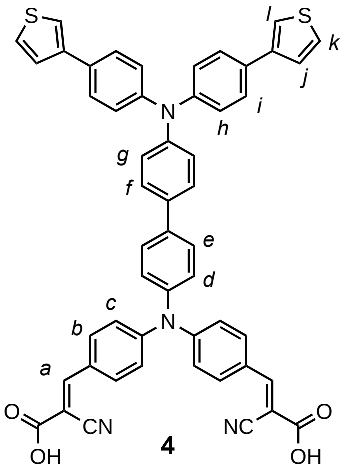

**4**

(3,3'-(((4'-(bis(4-(thiophen-3-yl)phenyl)amino)-[1,1'-biphenyl]-4-yl)azanediyl)bis(4,1-phenylene))bis(2-cyanoacrylic acid) (**4**)**:** To a solution of **14** (370 mg, 0.52 mmol) and cyanoacetic acid (250 mg, 2.94 mmol) in MeCN (25 mL) was added piperidine (0.1 mL) and the reaction mixture was then stirred and refluxed for 6 hrs (and the colour change from orange to red). After removing the solvent, the mixture was triturated with $CHCl_3$, to remove the starting materials affording the desired product as a red solid (280 mg, 0.33 mmol, 63.6%).$^{1}$H NMR (400 MHz,): δ = 8.01 (H*a*, s, 2H), 7.85 – 7.72 (H*b*, H*e*, m, 6H), 7.59 – 7.32 (H*f*, H*c*, H*l*, m, 8 H), 7.13 – 6.98 (H*d*, H*g*, H*h*, H*j*, H*k*, m, 12 H), 6.86 (H*i*, d, $^{3}J_{HH}$ = 8 Hz, 4H). HRMS (MALDI): m/z 842.20164 calculated for $C_{52}H_{34}N_2O_4S_2$: m/z 842.20215. *To help with 1H-NMR solubility, 2 drops of DMSO-d$^{6}$ was added. Poor solubility prevented the acquisition of 13C data.*

# 3. Summary of Physicochemical Characterization

**Table S1.** Physicochemical characterization of Bichromic Bipodal dyes.

| Compound | Code | ETPA $1_{ox}$ (V vs NHE)[a] | ETPA $2_{ox}$ (V vs NHE)[a] | UV-vis $\lambda_{max}$ *nm* (ε ×$10^4$ $M^{-1}cm^{-1}$) | |
|---|---|---|---|---|---|
| **L1 - OFc** | **L1** | 1.23 | | 483 (3.2)[b] | |
| **TPA$_2$CHO$_2$ - fc** | **6a** | 1.14 | 1.38 | 366 (3.9) | |
| **TPA$_2$Dye - ofc** | **1a** | 1.12 | 1.36 | 471 (3.6) | 407 (2.7) |
| **TPA$_2$thioCHO$_2$ - fc** | **9a** | 1.06 | 1.23 | 389 (5.7) | 304 (1.6) |
| **TPA$_2$thio Dye - Ofc** | **2a** | 1.04 | 1.18 | 421 (3.3) | |
| **TPA$_2$thio$_2$CHO$_2$ - fc** | **12a** | 1.02 | 1.15 | 397 (7.3) | |
| **TPA$_2$thio$_2$ Dye - ofc** | **3a** | 0.98 | 1.14 | 436 (1.5) | |
| **OMe$_2$TPA$_2$CHO$_2$ - fc** | **6b** | 0.93 | 1.39 | 373 (4.4) | |
| **OMe$_2$TPA$_2$ Dye - ofc** | **1b** | 0.89 | 1.36 | 470 (3.8) | 416 (3.2) |
| **OMe$_2$TPA$_2$thioCHO$_2$ - fc** | **9b** | 0.90 | 1.24 | 391 (4.1) | |
| **OMe$_2$TPA$_2$thio Dye – ofc** | **2b** | 0.86 | 1.19 | 433 (6.2) | |
| **OMe$_2$TPA$_2$thio$_2$CHO$_2$ - fc** | **12b** | 0.89 | 1.16 | 398 (4.5) | |
| **OMe$_2$TPA$_2$thio$_2$ Dye - ofc** | **3b** | 0.86 | 1.10 | 433 (0.27) | |
| **OHex$_2$TPA$_2$CHO$_2$ – fc** | **6c** | 0.91 | 1.39 | 373 (3.0) | 306 (1.2) |
| **OHex$_2$TPA$_2$Dye - ofc** | **1c** | 0.87 | 1.34 | 423 (4.9) | 302 (2.8) |
| **OHex$_2$TPA$_2$thioCHO$_2$ - fc** | **9c** | 0.88 | 1.23 | 391 (8.0) | |
| **OHex$_2$TPA$_2$thio Dye - ofc** | **2c** | 0.84 | 1.14 | 415 (3.4) | |
| **OHex$_2$TPA$_2$thio$_2$CHO$_2$ - fc** | **12c** | 0.88 | 1.16 | 397 (6.4) | |
| **OHex$_2$TPA$_2$thio$_2$ Dye - ofc** | **3c** | 0.84 | 1.10 | 437 (3.1) | |
| **SMe$_2$TPA$_2$CHO$_2$ - fc** | **6d** | 0.99 | 1.42 | 373 (6.4) | 329 (4.2) |
| **SMe$_2$TPA$_2$Dye – ofc** | **1d** | 0.98 | 1.39 | 471 (4.8) | 410 (3.9), 328 (4.2) |
| **SMe$_2$TPA$_2$thioCHO$_2$ - fc** | **9d** | 0.98 | 1.24 | 391 (6.4) | 332 (3.0) |
| **SMe$_2$TPA$_2$thio Dye - ofc** | **2d** | 0.94 | 1.21 | 428 (6.0) | 322 (3.5) |
| **SMe$_2$TPA$_2$thio$_2$CHO$_2$ - fc** | **12d** | 0.97 | 1.17 | 399 (8.7) | 331 (4.4) |
| **SMe$_2$TPA$_2$thio$_2$ Dye - ofc** | **3d** | 0.93 | 1.13 | 438 (5.2) | 326 (2.4) |
| **3Thio2 TPA$_2$CHO$_2$ – Fc** | **14** | 1.09 | 1.43 | | |
| **Thio2TPA2 Dye – Ofc** | **4** | 1.06 | 1.39 | 344 (3.3) | 469 (2.9), 399 (2.4) |

[a]Data collected using 0.1 M $NBu_4PF_6$ DCM solutions at 100 $mVs^{-1}$ and referenced to a ferrocene [Fc]/$[Fc]^+$ internal standard for the aldehyde precursors and Octamethylferrocene (OFc) [OFc]/$[OFc]^+$ internal standard for the furnished dyes. Calibrated vs. 0.700V for Fc and 0.225V for OFc. [b]value from previous work.[5]

# 4. UV-Vis and Fluorescence Spectroscopy

## UV-Vis in DCM

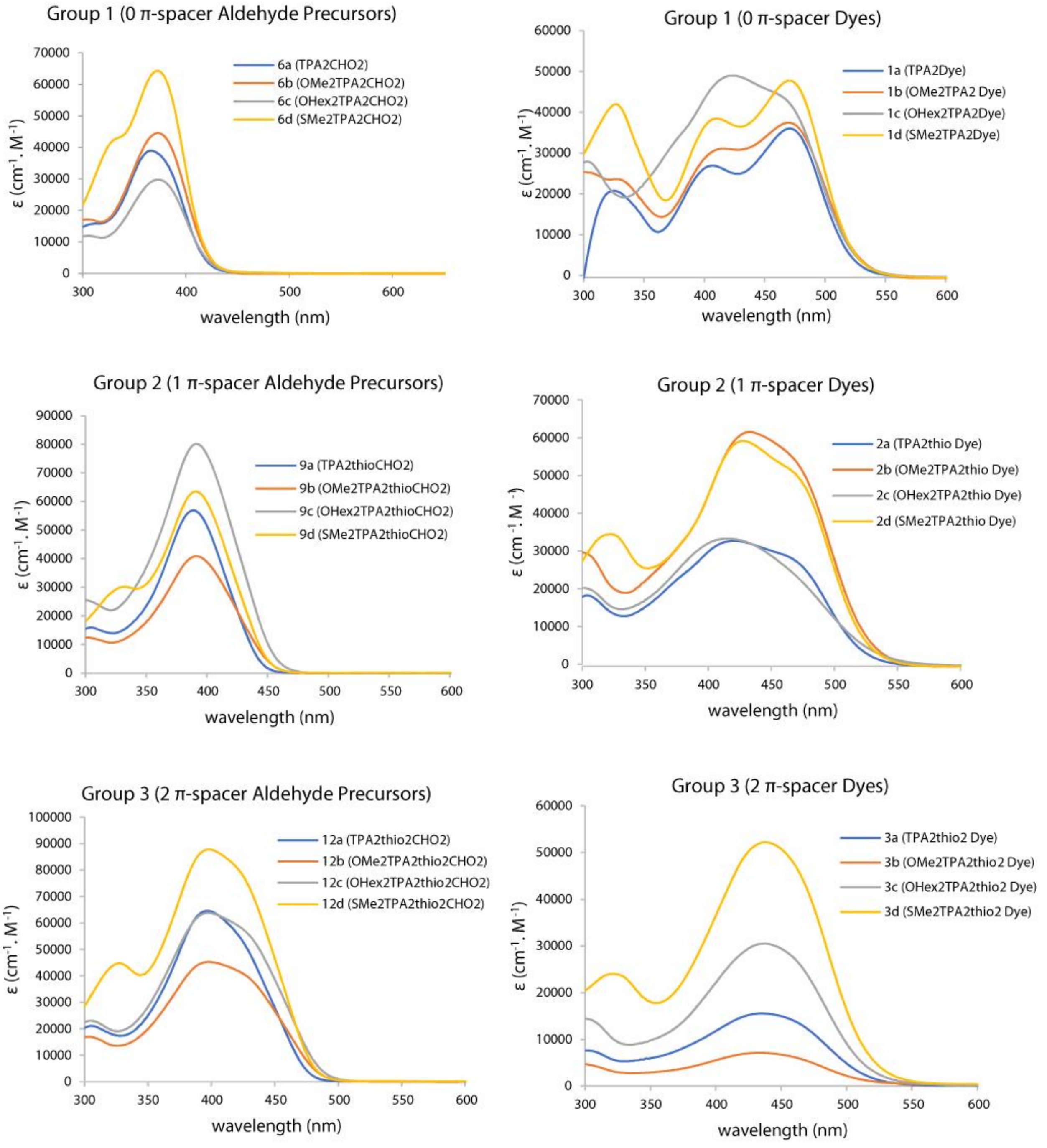


**Figure S1.** UV-vis absorption in DCM for dyes and their aldehyde precursors.

# Absorption and Fluorescence in DCM

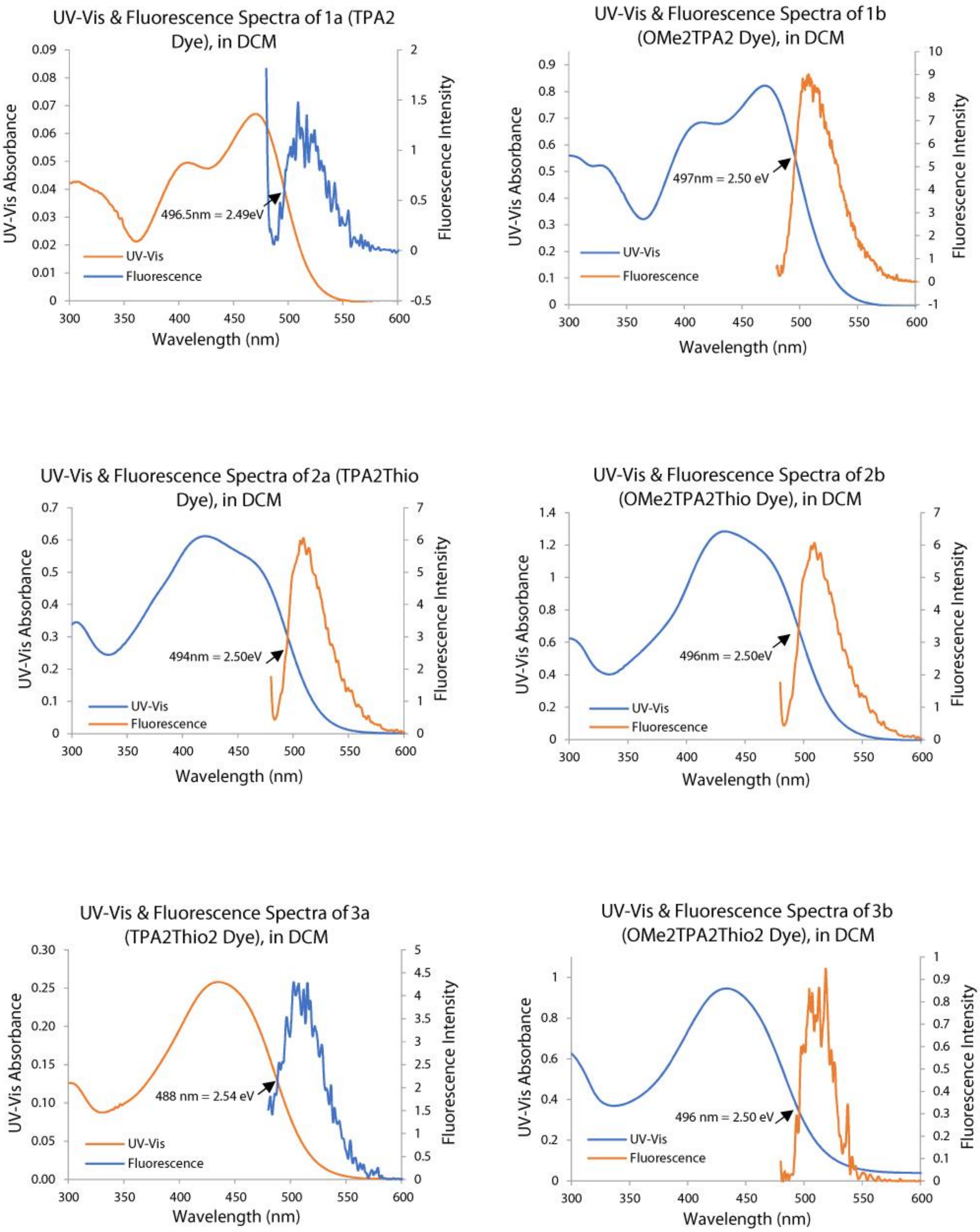


**Figure S2a.** UV-vis absorption and fluorescence spectra in DCM for dyes.

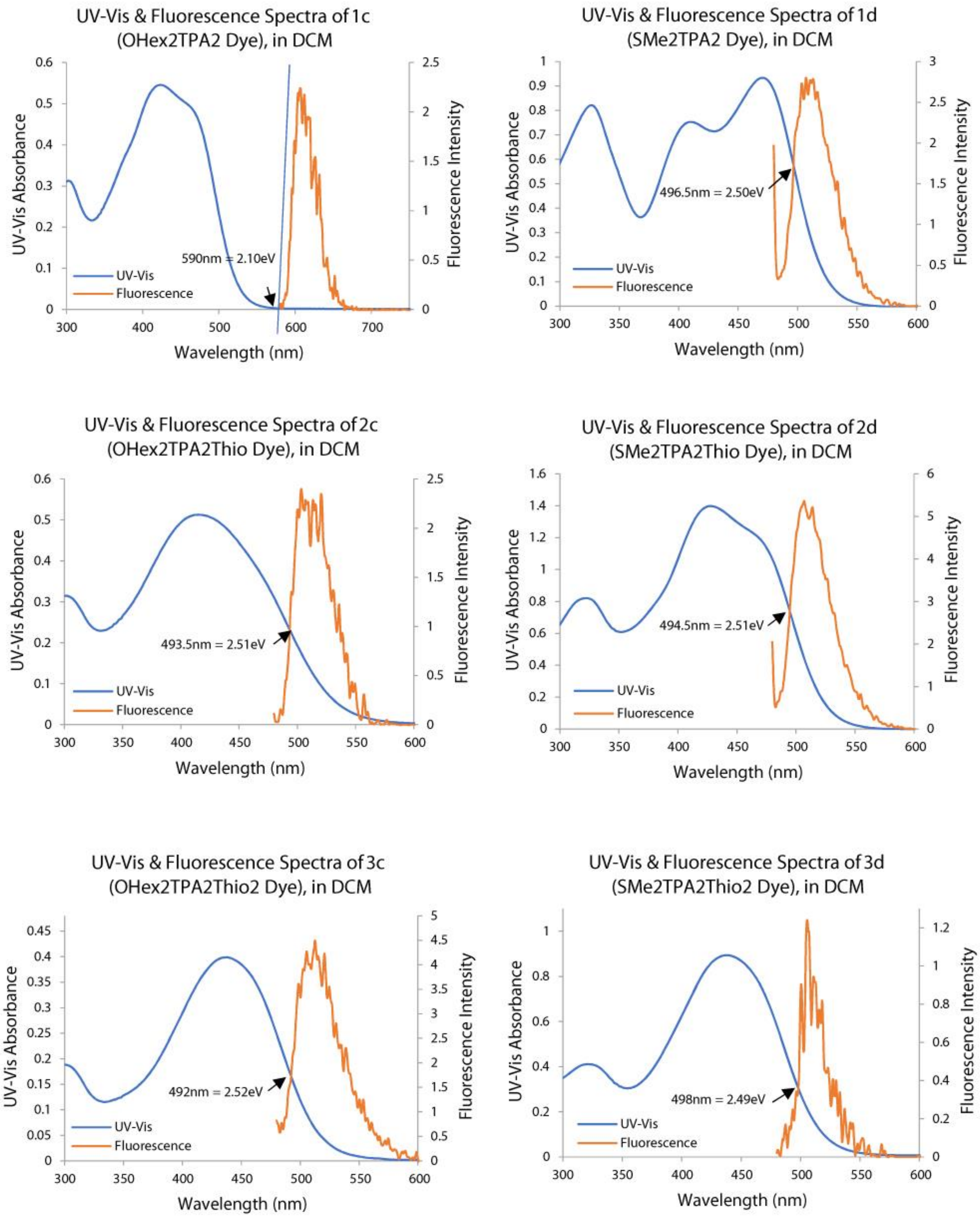


**Figure S2b.** UV-vis absorption and fluorescence spectra in DCM for dyes.

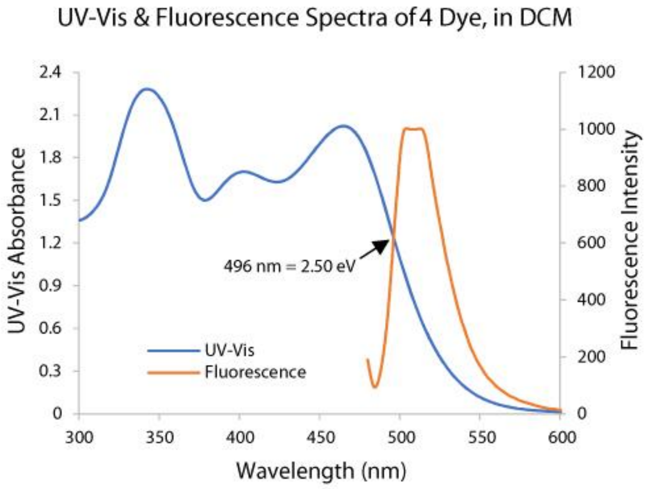


**Figure S2c.** UV-vis absorption and fluorescence spectra in DCM for dyes.

# 5. Cyclic Voltammetry

Cyclic Voltammetry of **6a** ($TPA_2CHO_2$-fc)

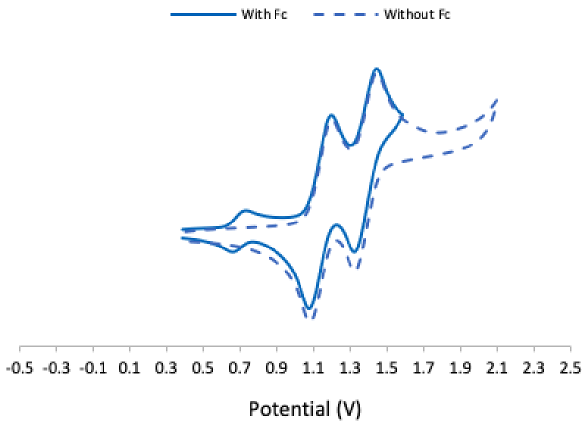


Cyclic Voltammetry of **1a** ($TPA_2$Dye-ofc)

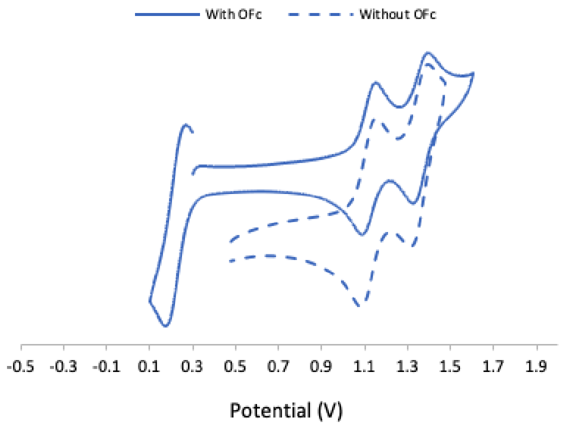


Cyclic Voltammetry of **9a** ($TPA_2$thio$CHO_2$-fc)

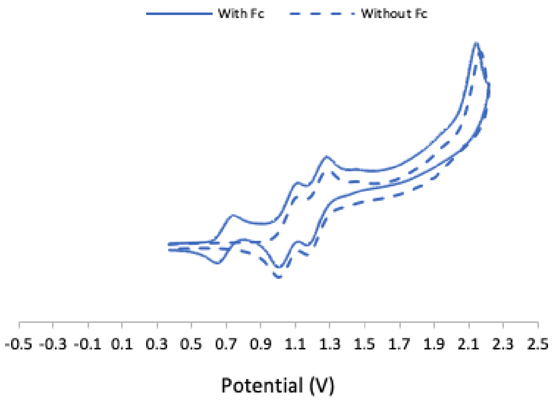


Cyclic Voltammetry of **2a** ($TPA_2$thio Dye-ofc)

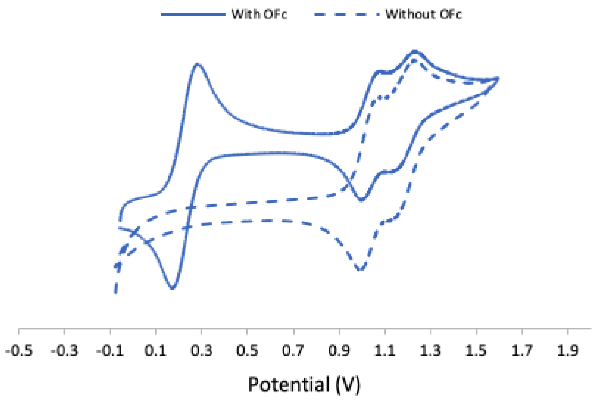


Cyclic Voltammetry of **12a** ($TPA_2thio_2CHO_2$-fc)

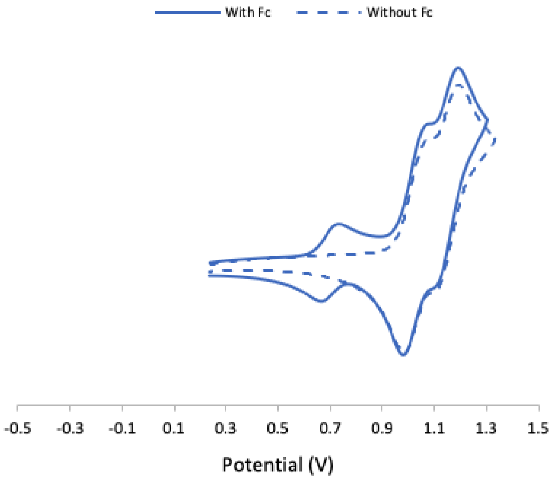


Cyclic Voltammetry of **3a** ($TPA_2thio_2$ Dye-ofc)

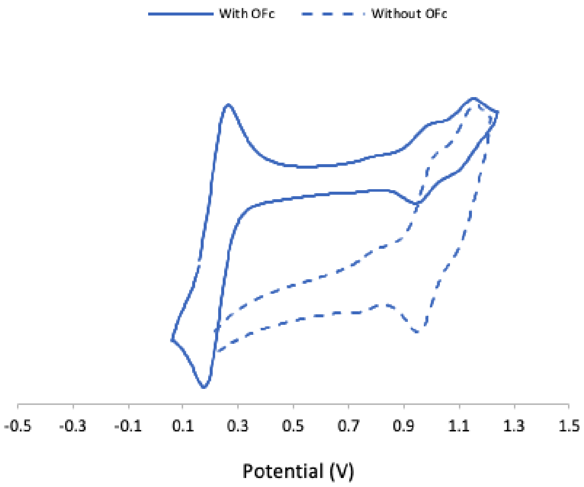

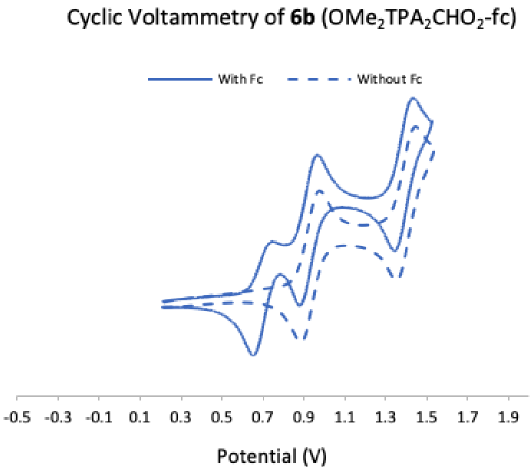
Cyclic Voltammetry of 6b (OMe2TPA2CHO2-fc)
With Fc
Without Fc
-0.5 -0.3 -0.1 0.1 0.3 0.5 0.7 0.9 1.1 1.3 1.5 1.7 1.9
Potential (V)

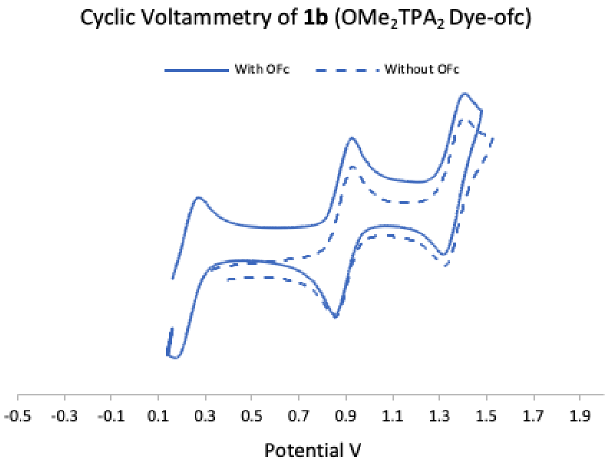
Cyclic Voltammetry of 1b (OMe2TPA2 Dye-ofc)
With OFc
Without OFc
-0.5 -0.3 -0.1 0.1 0.3 0.5 0.7 0.9 1.1 1.3 1.5 1.7 1.9
Potential V

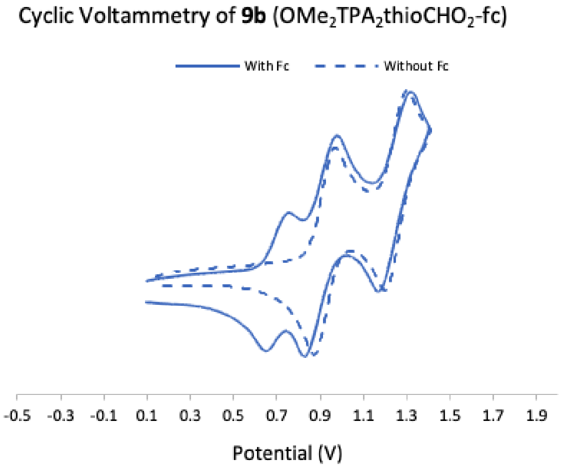
Cyclic Voltammetry of 9b (OMe2TPA2thioCHO2-fc)
With Fc
Without Fc
-0.5 -0.3 -0.1 0.1 0.3 0.5 0.7 0.9 1.1 1.3 1.5 1.7 1.9
Potential (V)

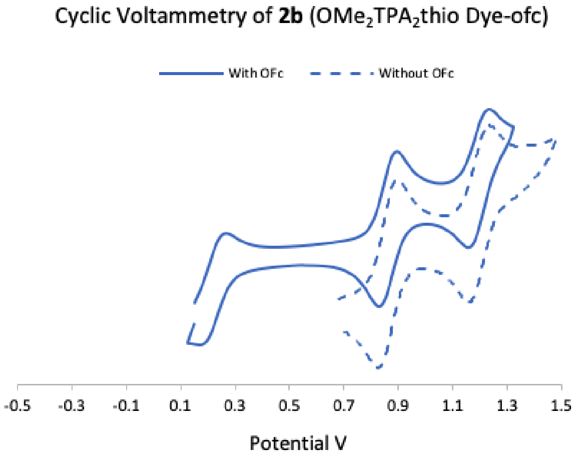
Cyclic Voltammetry of 2b (OMe2TPA2thio Dye-ofc)
With OFc
Without OFc
-0.5 -0.3 -0.1 0.1 0.3 0.5 0.7 0.9 1.1 1.3 1.5
Potential V

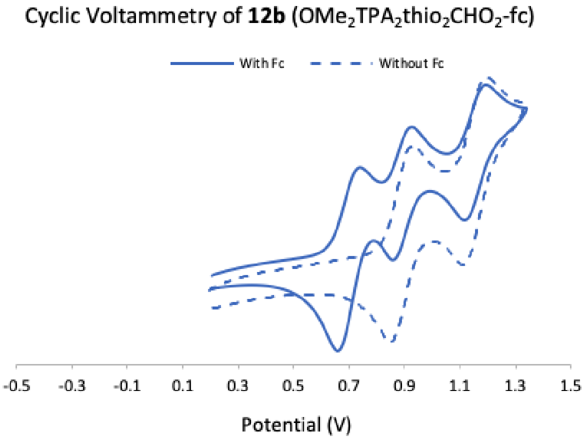
Cyclic Voltammetry of 12b (OMe2TPA2thio2CHO2-fc)
With Fc
Without Fc
-0.5 -0.3 -0.1 0.1 0.3 0.5 0.7 0.9 1.1 1.3 1.5
Potential (V)

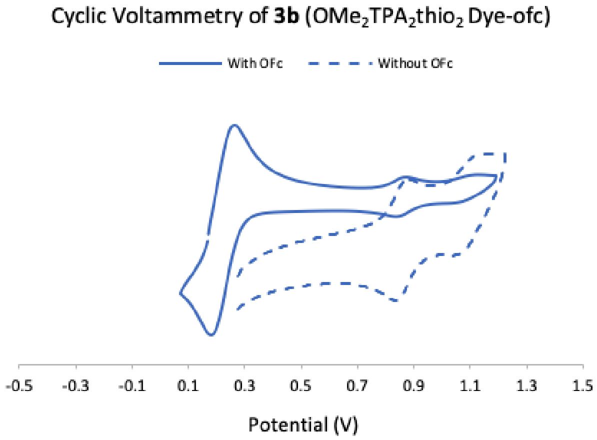
Cyclic Voltammetry of 3b (OMe2TPA2thio2 Dye-ofc)
With OFc
Without OFc
-0.5 -0.3 -0.1 0.1 0.3 0.5 0.7 0.9 1.1 1.3 1.5
Potential (V)

Cyclic Voltammetry of **6c** ($OHex_2TPA_2CHO_2$-fc)

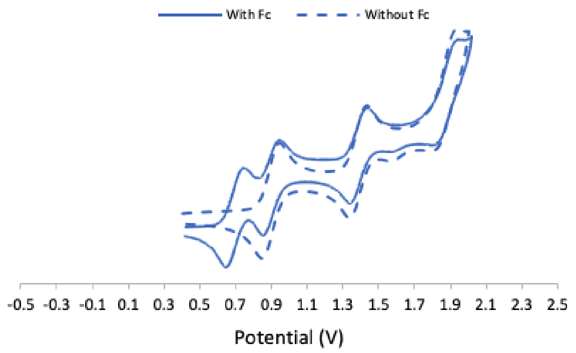


Cyclic Voltammetry of **1c** ($OHex_2TPA_2$ Dye-ofc)

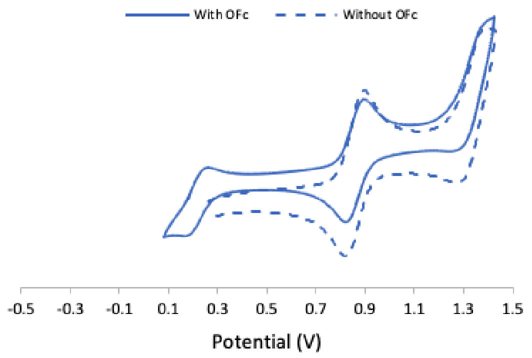


Cyclic Voltammetry of **9c** ($OHex_2TPA_2thioCHO_2$-fc)

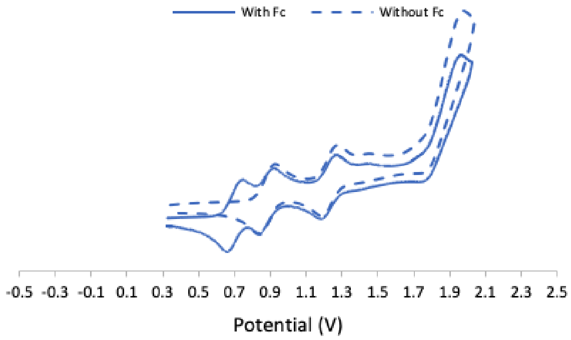


Cyclic Voltammetry of **2c** ($OHex_2TPA_2thio$ Dye-ofc)

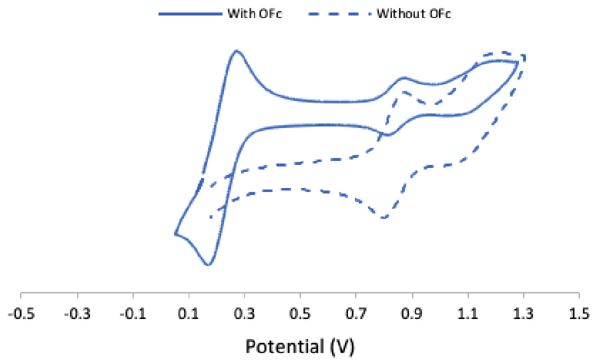


Cyclic Voltammetry of **12c** ($OHex_2TPA_2thio_2CHO_2$-fc)

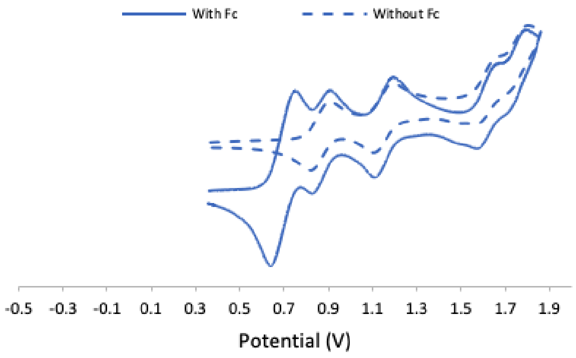


Cyclic Voltammetry of **3c** ($OHex_2TPA_2thio_2$ Dye-ofc)

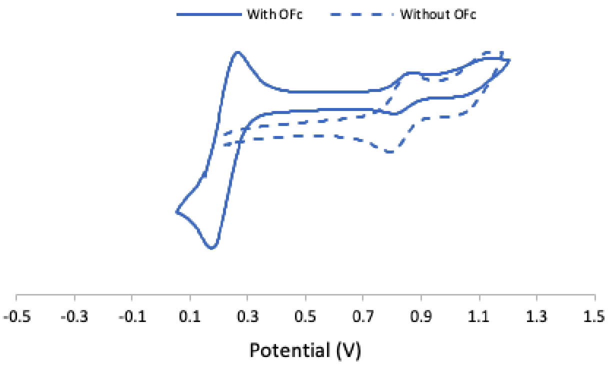

Cyclic Voltammetry of **6d** ($SMe_2TPA_2CHO_2$-fc)

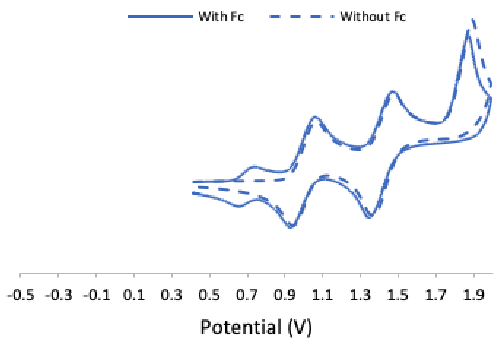


Cyclic Voltammetry of **1d** ($SMe_2TPA_2$ Dye-ofc)

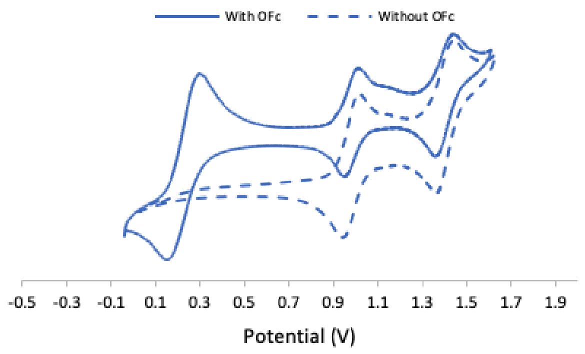


Cyclic Voltammetry of **9d** ($SMe_2TPA_2$thio$CHO_2$-fc)

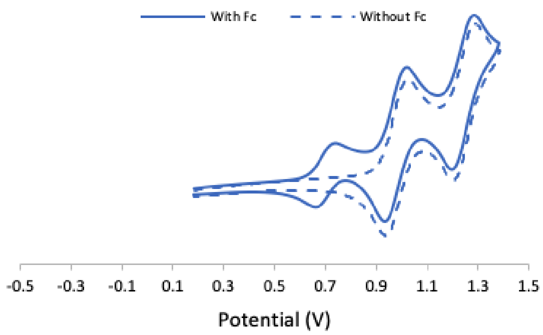


Cyclic Voltammetry of **2d** ($SMe_2TPA_2$thio Dye-ofc)

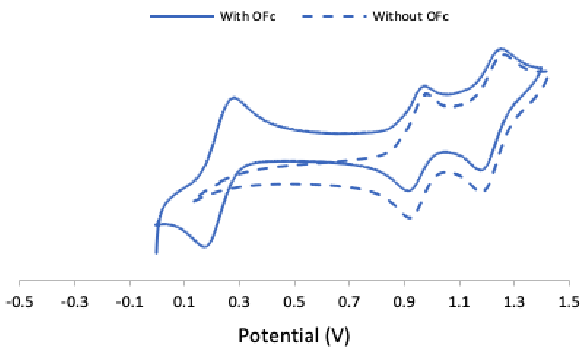


Cyclic Voltammetry of **12d** ($SMe_2TPA_2thio_2CHO_2$-fc)

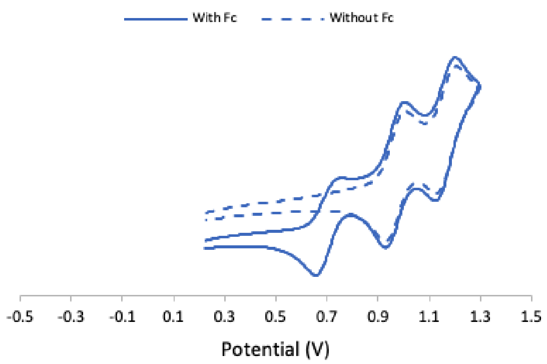


Cyclic Voltammetry of **3d** ($SMe_2TPA_2thio_2$ Dye-ofc)

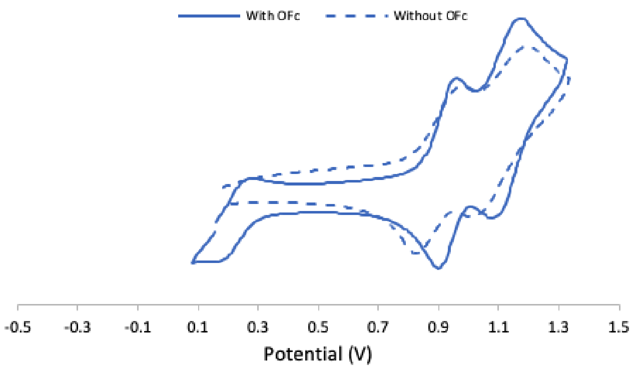

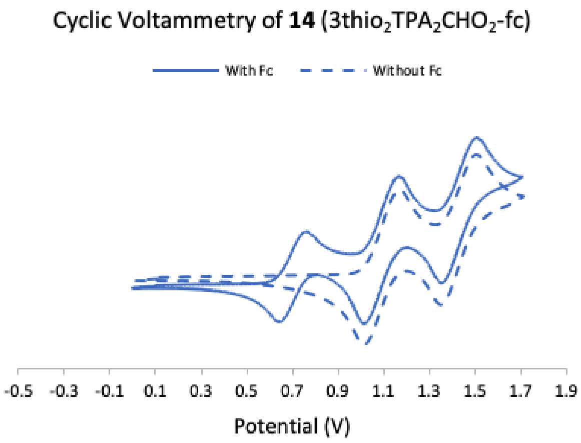
Cyclic Voltammetry of 14 (3thio2TPA2CHO2-fc)
With Fc
Without Fc
-0.5 -0.3 -0.1 0.1 0.3 0.5 0.7 0.9 1.1 1.3 1.5 1.7 1.9
Potential (V)

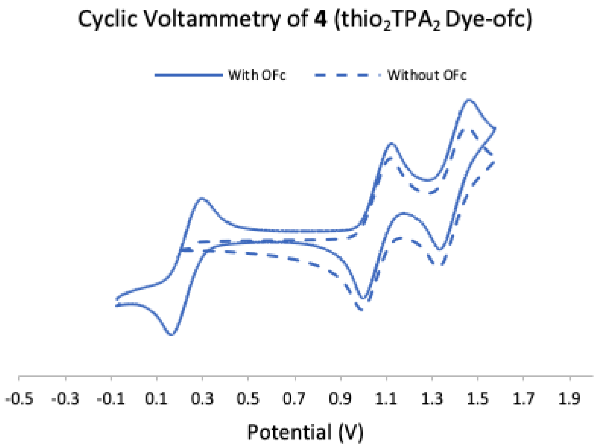
Cyclic Voltammetry of 4 (thio2TPA2 Dye-ofc)
With OFc
Without OFc
-0.5 -0.3 -0.1 0.1 0.3 0.5 0.7 0.9 1.1 1.3 1.5 1.7 1.9
Potential (V)

## 6. IV Curves

Best DSSC of **1a** (TPA2 Dye)

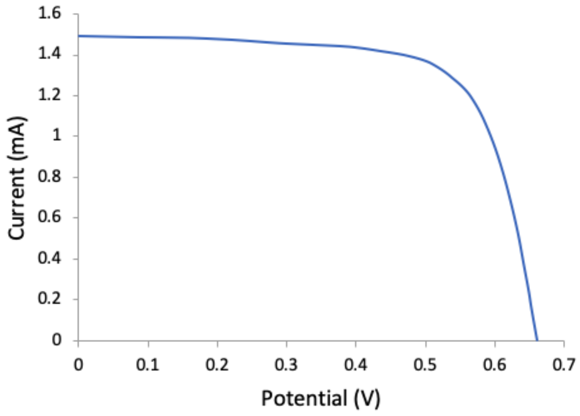


Best DSSC of **1b** (OMe2TPA2 Dye)

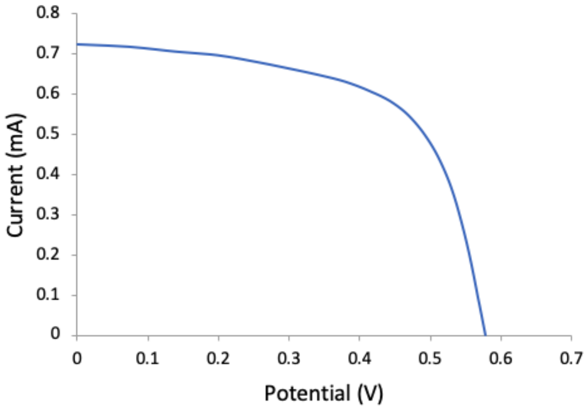


Best DSSC of **1c** (OHex2TPA2 Dye)

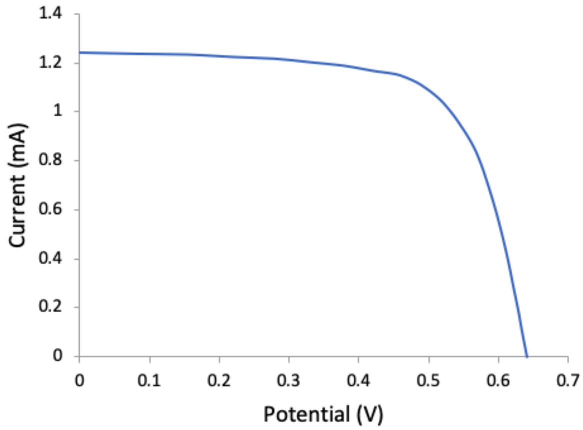


Best DSSC of **1d** (SMe2TPA2 Dye)

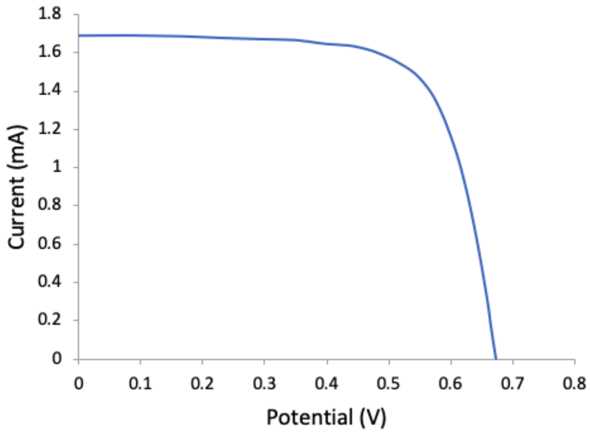


Best DSSC of **2a** (TPA2Thio Dye)

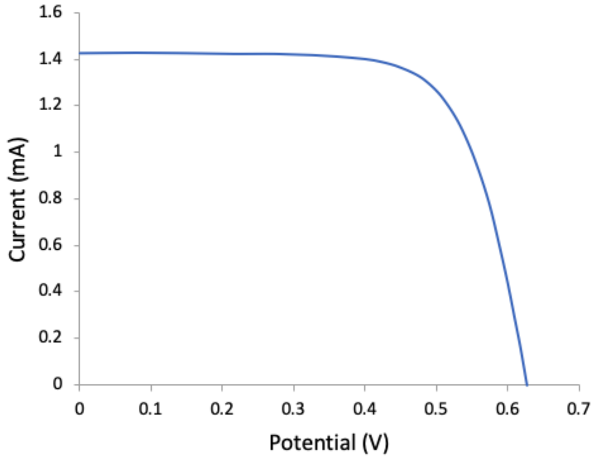


Best DSSC of **2b** (OMe2TPA2Thio Dye)

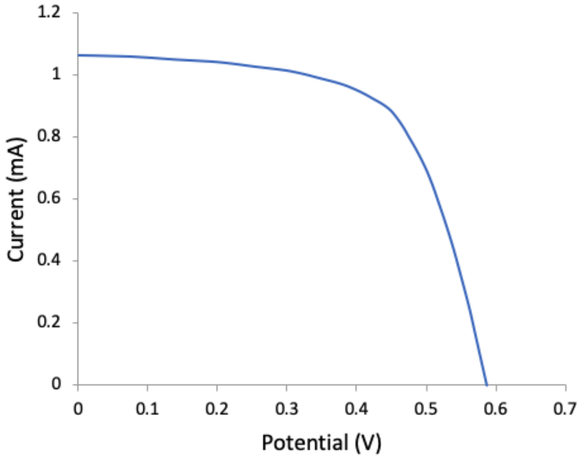

Best DSSC of **2c** (OHex2TPA2Thio Dye)

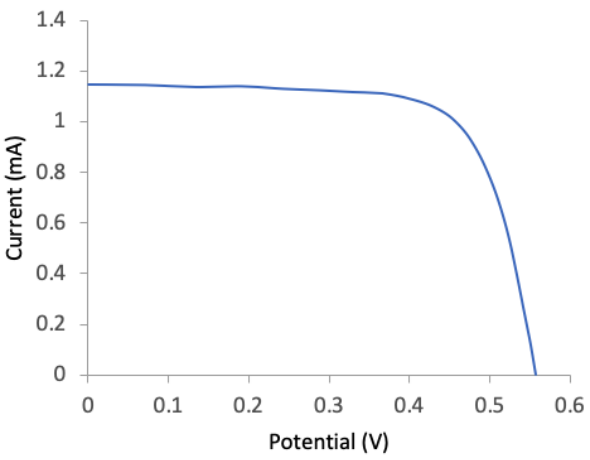


Best DSSC of **2d** (SMe2TPA2Thio Dye)

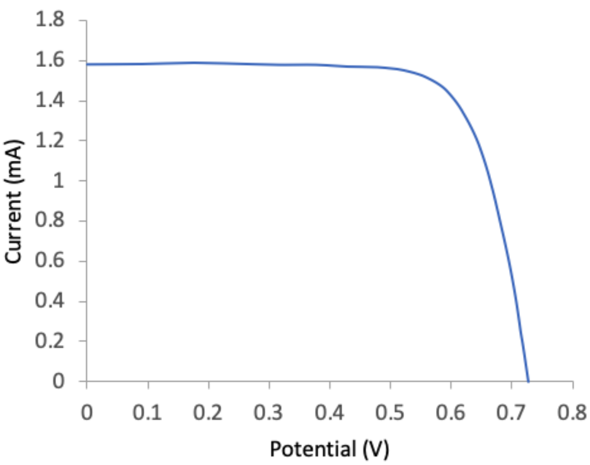


Best DSSC of **3a** (TPA2Thio2 Dye)

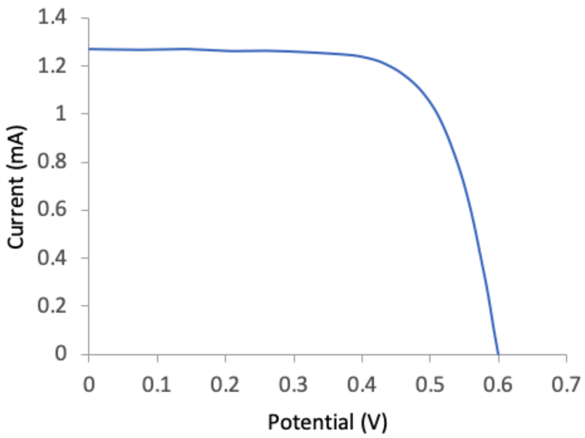


Best DSSC of **3b** (OMe2TPA2Thio2 Dye)

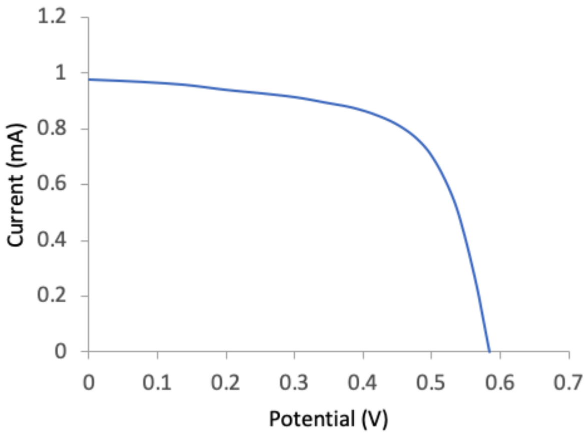


Best DSSC of **3c** (OHex2TPA2Thio2 Dye)

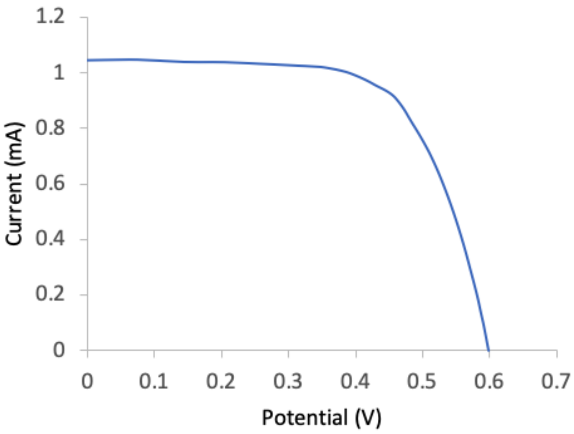


Best DSSC of **3d** (SMe2TPA2Thio2 Dye)

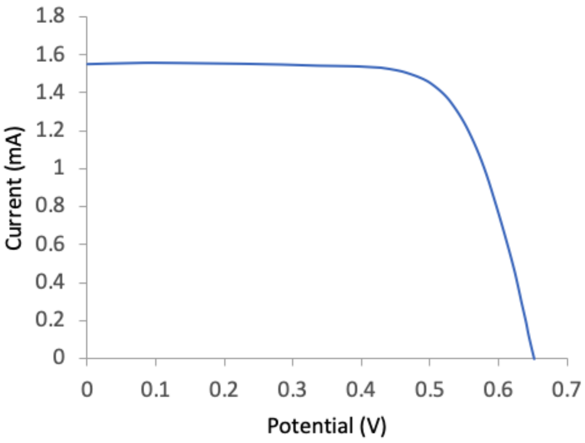

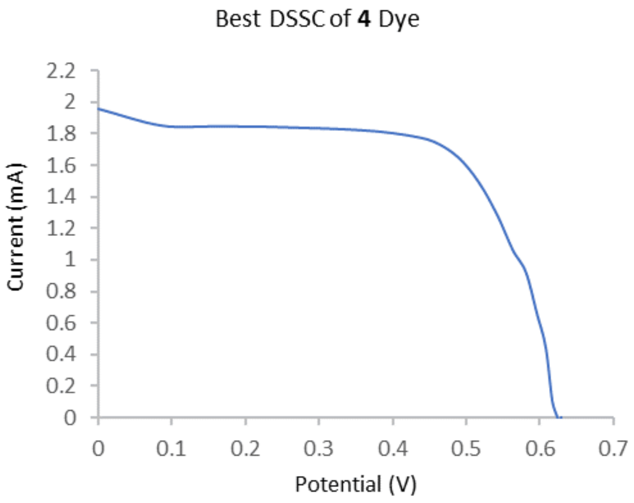


# 7. References


1. Fang, Y. *et al.* Phenylenevinylene copolymers of dihexylthienylbenzothiadiazole and triphenylamine or tetraphenylbenzidine: Synthesis, characterization and photovoltaic properties. *J. Mater. Sci.* **47**, 5706–5714 (2012).

2. Amthor, S. & Lambert, C. [2.2]Paracyclophane-bridged mixed-valence compounds: Application of a generalized mulliken-hush three-level model. *J. Phys. Chem. A* **110**, 1177–1189 (2006).

3. Ferdowsi, P. *et al.* Molecular Design of Efficient Organic D–A–Π –A Dye Featuring Triphenylamine as Donor Fragment for Application in Dye-Sensitized Solar Cells. *ChemSusChem* **11**, 494–502 (2018).

4. Yin, X. *et al.* Binary hole transport materials blending to linearly tune HOMO level for high efficiency and stable perovskite solar cells. *Nano Energy* **51**, 680–687 (2018).

5. Abdi, O. K. *et al.* Bipodal dyes with bichromic triphenylamine architectures for use in dye-sensitized solar cell applications. *RSC Adv.* **8**, 42424–42428 (2018).

6. Dienes, Y. *et al.* Selective tuning of the band gap of π-conjugated dithieno[3,2-6: 2′,3′-d] phospholes toward different emission colors. *Chem. - A Eur. J.* **13**, 7487–7500 (2007).

7. Bonnier, C., Machin, D. D., Abdi, O. K., Robson, K. C. D. & Koivisto, B. D. The effect of donor-modification in organic light-harvesting motifs: Triphenylamine donors appended with polymerisable thienyl subunits. *Org. Biomol. Chem.* **11**, 7011–7015 (2013).
